# Eco-Friendly Sample Preparation Trends for Exogenous Toxic Organic Compounds in Food: A Sustainable Perspective for LC-MS Analysis

**DOI:** 10.3390/foods15030517

**Published:** 2026-02-02

**Authors:** Mariel Cina, Alejandro Mandelli, María Del Valle Ponce, María Guiñez, Soledad Cerutti

**Affiliations:** 1 Instituto de Ciencias de La Tierra y Ambientales de La Pampa (INCITAP, CONICET-UNLPam), Facultad de Ciencias Exactas y Naturales, Universidad Nacional de La Pampa, Santa Rosa CP 6300, Argentina; cinamariel@gmail.com; 2 Consejo Nacional de Investigaciones Científicas y Técnicas (CONICET), Godoy Cruz 2290, Buenos Aires CP 1425, Argentina; alemandelli87@gmail.com (A.M.); mariadelvalleponce@gmail.com (M.D.V.P.); 3 Instituto de Química de San Luis (INQUISAL, CONICET-UNSL), Facultad de Química, Bioquímica y Farmacia, Universidad Nacional de San Luis, Laboratorio de Espectrometría de Masas, Bloque III, Ejército de Los Andes 950, San Luis CP 5700, Argentina; 4 Instituto de Física Aplicada (INFAP, CONICET-UNSL), Universidad Nacional de San Luis, Ejército de Los Andes 950, San Luis CP 5700, Argentina

**Keywords:** exogenous toxic compounds, food matrices, extraction techniques, LC-MS, green metrics, regulations

## Abstract

Exogenous toxic compounds in foods, arising from agricultural practices, environmental contamination, industrial processing, and packaging migration, remain a major global concern for food safety. These contaminants include mycotoxins, veterinary drug residues, antibiotics, pesticides, per- and polyfluoroalkyl substances, heterocyclic aromatic amines, and polycyclic aromatic hydrocarbons, which have multiple adverse effects on human and animal health. The continued presence of these substances highlights the need for reliable exposure assessment, strengthened regulatory frameworks, and advanced analytical methodologies. Food matrices introduce variability in analytical performance, making sample preparation a critical and often limiting step. Conventional extraction techniques such as solid-phase extraction, liquid–liquid extraction, and Quick, Easy, Cheap, Effective, Rugged, and Safe (QuEChERS) are still widely applied. Moreover, recent advances have highlighted sustainable alternatives aligned with the principles of green analytical chemistry. In this context, this review provides a comprehensive overview of recent advances (2020–2025) in environmentally friendly extraction techniques for determining exogenous toxic compounds in food samples analyzed by liquid chromatography coupled with mass spectrometry (LC–MS), including their sustainability. Special attention is given to the chemical nature and toxicological relevance of major exogenous organic contaminant families (specialized categories such as hormones and packaging-derived bisphenols were excluded due to distinct migration and metabolic pathways; however, these topics exceed the scope of this manuscript), the analytical challenges associated with different food matrices, and the evolution of extraction and cleanup techniques. Overall, this review integrates analytical robustness, matrix effects, and green metrics to support the development of reliable and more sustainable sample preparation strategies.

## 1. Introduction

The presence of exogenous toxic compounds in foods, whether introduced through agricultural practices, environmental contamination, industrial processing, or packaging migration, represents one of the most enduring challenges in food safety [1]. Exposure assessment, regulation, and analytical detection provide the foundation for understanding contaminant occurrence, evaluating potential health impacts, and implementing effective control measures to protect consumers [2]. Hazardous compounds include a wide range of chemical families, covering naturally occurring toxins, residues of veterinary drugs and antibiotics, pesticides, and industrial pollutants, as well as heat-induced contaminants [3,4]. These substances are critical targets for both research and routine monitoring due to their persistence, bioaccumulation, and well-established associations with carcinogenicity, endocrine disruption, and antimicrobial resistance [1,3,4]. In this context, analytical chemistry plays a central role in ensuring that food production systems remain safe and sustainable [5].

Liquid chromatography coupled to mass spectrometry (LC–MS) has emerged as an analytical workhorse for contaminant determination in food products [3,4,5]. The combination of ultra-high-performance liquid chromatography (UHPLC) with tandem (MS/MS) and high-resolution (HRMS) mass spectrometry provides the sensitivity and selectivity required to detect trace contaminants in complex food matrices [5]. Modern instruments routinely achieve ultra-low limits of detection and are capable of simultaneously screening hundreds of analytes [5]. Ongoing innovations, including improved ionization sources, chromatographic stationary phases, advanced acquisition modes, and data-processing algorithms, combined with food-focused, data-driven screening, have expanded the analytical capabilities of LC–MS-based workflows [4]. HRMS platforms such as Orbitrap and QTOF systems now enable both suspect and non-targeted screening, allowing laboratories to track regulated compounds while identifying previously unknown and unexpected contaminants.

However, powerful instrumentation cannot compensate for inadequate sample preparation. Food matrices are chemically complex systems whose composition can significantly influence analyte recovery and signal response [6,7]. Consequently, the efficiency of extraction and cleanup steps largely determines analytical performance [6,7,8,9,10,11]. It is therefore crucial to understand how the physicochemical nature of the matrix affects analyte behavior to design reliable and reproducible methods [6,7,8,9,10,11]. Traditionally, extraction in food analysis has relied on well-established approaches such as solid-phase extraction (SPE), liquid–liquid extraction (LLE), and Quick, Easy, Cheap, Effective, Rugged, and Safe (QuEChERS), which combine features of both SPE and LLE [2,11]. These methods continue to be fundamental for the analysis of contaminants due to their robustness and wide applicability [6,7,8,9,10,11].

Nevertheless, the increasing emphasis on sustainability and laboratory safety has driven a shift toward eco-friendly extraction methods that minimize solvent use, reduce waste, and adopt greener materials without compromising analytical performance [6,7,12]. This evolution is closely aligned with the principles of green analytical chemistry (GAC) and Green Sample Preparation (GSP) [7,8,9,10,12,13]. Over the past few years, the assessment of the environmental and operational impact of these new workflows has relied on several green metrics that integrate ecological, analytical, and practical dimensions, offering quantitative means to compare and optimize methods [8,9,10,13,14,15]. However, the systematic application of such metrics in food contaminant analysis remains limited [9,10,12,13].

This review systematically evaluates advancements between 2020 and 2025 in eco-friendly sample preparation for the LC–MS analysis of exogenous toxic organic compounds in food matrices. Adopting the principles of green sample preparation as an operational framework, and adopting the reported metrics, this work critically assesses the interplay between contaminant physicochemical properties and the structural complexity of plant-based, animal-based, and processed matrices. A data-driven comparison between conventional techniques and emerging sustainable workflows is provided. Furthermore, this review bridges the gap between green pre-analytical stages and high-resolution instrumental, ensuring a holistic evaluation of analytical performance metrics—such as sensitivity and selectivity—relative to the environmental footprint of the entire workflow.

## 2. Exogenous Toxic Compounds in Food: Chemical Families, Toxicological Relevance, and Regulatory Frameworks

Food products can contain toxic contaminants of diverse origin. They are either naturally occurring, generated during processing, or introduced through anthropogenic activities. Understanding their chemical nature is essential to explain their persistence, mobility, and potential health effects, while their reliable detection requires selective analytical methodologies tailored to their physicochemical properties. In recent years, international monitoring programs and regulatory frameworks have emphasized the need for harmonized criteria for control and risk assessment to ensure global food safety. This section provides an overview of the main chemical families of exogenous toxic compounds of relevance in food matrices, summarizing their origin, structure, toxicological significance, and current regulatory context.

### 2.1. Mycotoxins

Mycotoxins are toxic secondary metabolites produced mainly by fungi of the *Aspergillus*, *Penicillium*, and *Fusarium* genera. The most relevant compounds include aflatoxins (B1, B2, G1, G2, and M1), ochratoxin A (OTA), fumonisins, deoxynivalenol (DON), and zearalenone (ZEA), which are widely detected in cereals, nuts, and dairy products [16,17,18,19]. Structurally, many of these molecules possess polycyclic backbones bearing lactone and/or phenolic groups that confer high thermal stability and resistance to degradation during processing [20,21]. The recognition of mycotoxins as a major food-safety issue dates to the 1962 “Turkey X disease” outbreak, which was later attributed to peanut meal contaminated with aflatoxins. Since then, contamination has been acknowledged as a global challenge affecting a substantial proportion of food commodities [16,17,18,19]. From a toxicological perspective, several mycotoxins are classified by the International Agency for Research on Cancer (IARC) as carcinogenic (Group 1), probably carcinogenic (Group 2A), or possibly carcinogenic (Group 2B) [22,23,24]. Co-exposure is common and may yield additive or synergistic effects, complicating risk assessment [25]. In addition, “modified” or “masked” forms (e.g., DON-3-glucoside), produced by plant defense mechanisms, can be hydrolyzed in the gastrointestinal tract and release the parent toxin, thereby increasing overall exposure that is often underestimated in routine monitoring [2,26]. Current international frameworks (e.g., EU and Codex) set strict maximum levels for major mycotoxins in food—typically in the μg kg^−1^ range—with even more restrictive limits for infants and for dairy-based products (Table S1, related references [1,2,3,4,5,6,7,8,9,10,11,12,13,14,15,16,17,18,19,20,21,22,23,24,25,26,27,28,29,30,31,32,33,34,35,36] are also cited in the Supplementary Materials) [27,28]. As mentioned, these molecules are difficult to remove and extract, primarily because they possess high thermal stability and resist degradation. Additionally, their moderate-to-high polarity, particularly in “masked” or modified forms like DON-3-glucoside, require extraction solvents that can effectively compete with matrix interferences like sugars and starches in cereals. In lipid-rich matrices like vegetable oils, the lipophilic nature of the matrix complicates the partitioning of specific mycotoxins, often resulting in unacceptably low recoveries (less than 50%) and necessitating advanced cleanup to avoid signal suppression.

### 2.2. Veterinary Drugs

Veterinary drugs include antiparasitic, anti-inflammatory, antibiotic, and hormonal agents used in livestock, aquaculture, and animal husbandry for therapeutic and prophylactic purposes. Their structural diversity governs absorption, metabolism, and persistence in tissues (notably liver, kidney, and fat), influencing bioaccumulation and transfer along the food chain [29,30,31]. Residues in food raise public health concerns due to potential allergic reactions, endocrine disruption, chronic toxicity, and genotoxicity; moreover, misuse contributes to antimicrobial resistance (AMR), a critical global threat. International and regional frameworks (EU, Codex, and WOAH—the World Organization for Animal Health-) establish maximum residue limits (MRLs) typically in the μg kg^−1^ range and define reference points for action (RPAs) for non-authorized substances (Table S1) [32,33,34,35,36]. Detections of banned sedatives or antivirals in aquaculture products illustrate the need for sustained surveillance. Thermal processing and binding reactive metabolites to tissue proteins can alter measured residue levels, with practical implications for exposure assessment [37]. Coordinated monitoring and sustainable production practices—under the One Health approach—are essential to minimize exposure and curb AMR [38,39,40].

Consideration is directed toward antibiotics, which represent one of the most rigorously regulated categories of veterinary drugs due to their extensive therapeutic and prophylactic applications in food-producing animals. Major families include β-lactams, macrolides, tetracyclines, sulfonamides, and quinolones, which differ in polarity, stability, and affinity for tissue proteins or lipids (Table S1). Residues may result from legitimate treatments or from unregulated practices (e.g., growth promotion) and are linked to the emergence and dissemination of AMR in pathogenic bacteria [41,42,43,44]. Regulatory authorities (e.g., EU, Codex) set matrix-dependent MRLs typically in the μg kg^−1^ range; zero-tolerance policies apply to specific compounds such as chloramphenicol and nitrofurans due to recognized genotoxicity [32,33]. Given the complexity of high-protein and high-fat matrices, robust extraction/cleanup is indispensable for reliable quantification (e.g., LLE, SPE, and microextraction strategies) [41,42,43,44]. The broad range of pKa values and charge states (e.g., in fluoroquinolones) complicates the optimization of a universal extraction pH. Furthermore, high affinities for tissue proteins in animal matrices require aggressive deproteinization steps (e.g., using acetonitrile or trichloroacetic acid) to release bound residues before analysis.

### 2.3. Non-Polar Pesticides

Pesticides are used widely to protect crops and enhance yields, especially in fruits, vegetables, and cereals. Lipophilic groups—chiefly organochlorines, organophosphates, and synthetic pyrethroids—are of particular concern due to persistence and bioaccumulation potential; several are recognized as persistent organic pollutants (POPs) [45,46,47]. Compounds such as DDT, aldrin, dieldrin, chlorpyrifos, and parathion exhibit high lipid solubility and low degradability, favoring accumulation in fatty tissues and biomagnification through food chains. Chronic exposure has been associated with endocrine disruption, neurotoxicity, reproductive effects, and carcinogenicity. Lipid-rich matrices also generate strong matrix effects during instrumental analysis, underscoring the need for selective cleanup [47]. At the regulatory level, EU frameworks define commodity-specific MRLs generally within the μg kg^−1^ to mg kg^−1^ range; more stringent criteria apply to infant and organic foods (Table S1). Furthermore, EU method-validation guidance promotes harmonized, selective multiresidue procedures, and several legacy pesticides are controlled internationally under the Stockholm Convention on POPs [48,49]. When extracting these compounds, it is important to address the co-extraction of natural lipids, as these can lead to significant matrix effects and may contaminate the ion sources of mass spectrometers. To achieve effective purification, selective sorbents such as C18 or PSA are needed to separate the target analytes from the fatty matrix while maintaining good recovery rates.

### 2.4. Per- and Polyfluoroalkyl Substances

Per- and Polyfluoroalkyl Substances (PFASs) constitute a large family of synthetic organofluorines characterized by exceptionally strong C–F bonds that confer environmental persistence and resistance to remediation. Prominent members—PFOA, PFOS, PFNA, and PFHxS—are listed as POPs and have been used in non-stick coatings, firefighting foams, and water-repellent materials, enabling broad environmental dissemination and detectability in soils, water, and food [50,51,52]. Their chemical stability and amphiphilicity favor bioaccumulation and trophic transfer [53]. Epidemiological evidence links PFAS exposure to hepatotoxicity, endocrine disruption, dyslipidemia, and potential carcinogenicity, even at low, sustained levels [50,51,52]. EFSA established a tolerable weekly intake (TWI) for the sum of PFOA, PFOS, PFNA, and PFHxS, and the EU has introduced maximum levels for selected foodstuffs, particularly of animal origin—generally in the ng g^−1^ (μg kg^−1^) order (Table S1) [54,55]. Although legacy compounds face restrictions, replacement PFASs (e.g., GenX and F-53B) have raised new toxicological concerns, reinforcing the need for global monitoring and safer alternatives [56,57]. Unlike many organic pollutants that partition solely into lipids, PFASs exhibit a strong affinity for proteins. This necessitates specialized extraction protocols that can disrupt protein binding while managing the widespread environmental persistence that often leads to background contamination during laboratory processing.

### 2.5. Heterocyclic Aromatic Amines

Heterocyclic Aromatic Amines (HAAs) are nitrogen-containing compounds formed during the heating of protein-rich foods (meats, fish). They rank among the most potent mutagens and are implicated in carcinogenesis [58,59,60]. HAAs typically occur at ng g^−1^ to μg kg^−1^ levels, with formation driven by temperature–time profiles and precursor availability (creatine, amino acids, reducing sugars) via Maillard and pyrolytic pathways (≥150 °C). Two major classes are recognized—aminoimidazoarenes (e.g., IQ, MeIQx) formed at moderate temperatures and aminocarbolines (e.g., PhIP, AαC) prevalent at >300 °C [58,59,60]. Beyond free forms, HAAs can exist in a form that is covalently bound to proteins; digestive hydrolysis may release free HAAs, increasing bioavailability and toxic potential [61,62]. International frameworks (e.g., EU, EFSA, Codex) recognize HAAs as contaminants of concern in heat-processed foods and monitor them at ng g^−1^ to μg kg^−1^ levels (see Table S1 in the Supplementary Materials). One of the analytical challenges is their presence in highly complex matrices, like plant-based milks or cooked meats, necessitating high enrichment factors. The proteins and fats present require thorough phase separation and cleanup to avoid interference with trace-level detection.

### 2.6. Polycyclic Aromatic Hydrocarbons

Polycyclic Aromatic Hydrocarbons (PAHs) and their nitrated (NPAHs) and oxygenated (OPAHs) derivatives are ubiquitous contaminants mainly associated with incomplete combustion and high-temperature processing (smoking, grilling, and roasting) [63,64,65]. Their fused aromatic-ring structures confer lipophilicity and persistence, supporting accumulation in fatty matrices and biomagnification. Many PAHs are mutagenic and carcinogenic; benzo[a]pyrene (BaP) is a Group 1 carcinogen and an indicator of PAH contamination. Several NPAHs/OPAHs show higher direct mutagenicity than their parents and some hydroxylated OPAHs are labile in aqueous media, complicating measurement and risk assessment [63,66,67]. Regulatory schemes identify a core set of PAHs for monitoring and set maximum levels—typically in the low μg kg^−1^ range—using BaP and the PAH4 sum (BaA, Chr, BbF, BaP) as markers in relevant commodities (Table S1) [68]. Despite advances in analysis and mitigation (process control, antioxidants), the persistence and transformation of PAHs and derivatives warrant continuous monitoring. In matrices like smoked honey or edible oils, the high sugar or lipid content directly hinders the release of PAHs. Their low solubility in aqueous phases requires dissolution in organic solvents followed by rigorous cleanup to achieve the sensitivity needed for regulatory compliance.

### 2.7. Regulatory Constraints and Analytical Challenges

The contaminant families described in this section exhibit pronounced structural and physicochemical variability that strongly influences extraction efficiency, analyte stability, and ionization response, all of which are essential for reliable LC–MS determination. The regulatory limits, as summarized in Table S1, range from μg kg^−1^ for mycotoxins and veterinary residues, to mg kg^−1^ tolerances for non-polar pesticides, and ng g^−1^ for PFASs and thermally derived PAHs/HAAs. These diverse concentration thresholds highlight the need for analytical workflows capable of achieving trace-level detection while maintaining rigorous control over matrix effects. In addition, regulatory and analytical constraints underline the importance of matrix-specific extraction strategies, selective cleanup to reduce co-extracted interferents, and the integration of advanced LC–MS/HRMS methodologies to enable reliable multiresidue monitoring across chemically diverse food contaminants. This technical foundation establishes the basis for the matrix-based analytical challenges addressed in the following sections.

## 3. Food Matrices’ Impact on the Occurrence of Exogenous Toxic Compounds

The detection of exogenous contaminants in food poses significant analytical challenges due to the complex structural and chemical nature of food matrices. Plant-based matrices contain pigments and starches that impede the extraction of lipophilic compounds, whereas animal-based matrices, which are rich in lipids and proteins, require efficient methods for residue release. These challenges are intensified in processed foods that combine both plant and animal components. The extensive physicochemical diversity of the exogenous contaminants described above delineates not only a broad toxicological spectrum but also the challenge of monitoring and overcoming the interactions of these compounds with the molecular environment of each food. This requires protocols capable of operating in chemically complex matrices subjected to multiple manufacturing stages, thereby requiring the development and application of suitable extraction and cleanup techniques [69,70,71,72,73,74,75,76,77,78]. The distribution of publications reporting major classes of exogenous toxic compounds across different food matrices is depicted in Figure S1.

### 3.1. Plant Matrices

Plant-based foods, including fruits, vegetables, cereals, legumes, and nuts, are rich in nutrients and bioactive compounds essential for human health. Analytically, these matrices combine nutritional diversity with chemical variability, shaping extraction behavior. Their composition and environmental exposure make them prone to contamination with exogenous toxic substances, including mycotoxins, pesticides, antibiotics, polyfluoroalkyl substances, and veterinary drugs, as well as derivatives of polycyclic aromatic hydrocarbons and heterocyclic aromatic amines [71]. These contaminants bioaccumulate from the environment or become incorporated into food matrices at various stages of the food production chain. Analysis of these matrices has intensified over the past five years, with a particular focus on pesticide residues and mycotoxins [79,80]. Mycotoxins commonly contaminate matrices such as cereals and flour (e.g., rice, corn), representing one of the most prevalent chemical concerns. Their presence is associated with the growth of filamentous fungi (mainly *Fusarium*, *Aspergillus*, and *Penicillium* species) that produce these toxic secondary metabolites under favorable conditions of temperature and humidity during harvesting or storage. The persistence of mycotoxins such as patulin in apple juice or aflatoxin B1 in rice is due to the chemical stability of these low-molecular-weight molecules during processing. Beyond the lipophilic nature of these contaminants, the oil matrix itself presents a lipid-rich environment that complicates selective extraction due to co-elution issues [81,82,83,84,85,86,87,88]. The continuous assessment of pesticide residues in food matrices, such as fruits and vegetables, remains a key area of study due to the intensive use of these chemicals in agriculture and the potential for their mismanagement in crop protection. The behavior and persistence of pesticides in food are governed not only by their lipophilicity but also by matrix-related processes, including sorption within plant tissues, diffusion dynamics, and abiotic factors such as temperature and moisture, which together determine residue retention levels [88]. Polycyclic aromatic hydrocarbons and their derivatives, due to their strong lipophilicity, preferentially partition into lipid-rich matrices. Thus, these compounds have been detected in products such as cereals, vegetables, and smoked tea [86,89,90]. Per- and polyfluoroalkyl substances pose an increasing threat to plant-derived foods because they can enter agricultural systems through contaminated irrigation water, biosolids, industrial emissions, or atmospheric deposition. This inherent resistance to thermal decomposition also explains their occurrence in edible oils, where PFASs can accumulate through environmental uptake, adsorption to lipid fractions, or migration from food-contact materials [91].

Plant-based beverages represent an emerging area of research due to their growing popularity. Concentrations of HAAs have been reported in plant-based drinks based on almond, soy, cashew, and peanut [89]. From an analytical perspective, these matrices (which are rich in lipids, proteins, and sugars) undergo intensive heat treatments that promote the formation or deposition of these compounds.

In summary, plant matrices play a dual role in food safety; while they provide essential nutrients and bioactive compounds, their complex physicochemical composition makes them highly susceptible to contamination and retention of exogenous toxic compounds.

### 3.2. Animal Matrices

The complexity of animal matrices governs the behavior, retention, and transformation of exogenous toxic compounds. The above-mentioned characteristics promote bioaccumulation and transfer along the food chain, highlighting the need for strengthened food safety monitoring and mitigation strategies. The most studied animal matrices include milk, meat, eggs, honey, and dairy products, where antibiotics, veterinary drugs, pesticides, and PFASs are the primary focus. Milk and dairy products, due to their widespread consumption, are one of the most studied matrices to ensure the absence of residues of veterinary drugs and antibiotics in this matrix [91,92,93,94,95,96,97]. Their presence results from their extensive use in animal husbandry for therapeutic, prophylactic, or growth-promoting purposes. Residues exceed the MRLs mainly due to failure to comply with withdrawal periods before slaughter or milking or due to inappropriate dosing [98]. These residues often have a high octanol/water partition coefficient (for example, chloramphenicol, organophosphate pesticides, and parasiticides like ivermectin), which are lipophilic and accumulate in adipose tissue, which is an important reservoir, releasing these compounds over the long term. Specific veterinary drugs illustrate these challenges; for instance, imidocarb, used in cattle, requires a long withdrawal period because it binds strongly to various tissues, such as muscle tissue [69,72,97,98,99,100,101,102]. In addition, residues of veterinary medicines, antibiotics, and pesticides are frequently detected in matrices such as milk and eggs [103]. Due to their widespread environmental presence and extensive use in agriculture, factors such as water runoff and agricultural practices facilitate their transfer into animals and their subsequent presence in food products. The presence of enrofloxacin has been reported, although its use has been banned in poultry; its presence in eggs is still a reality, as residues have been detected in fresh eggs [103]. On the other hand, PFASs are found in animal products, particularly in aquatic foods such as fish, oysters, and crabs, as well as in terrestrial sources including milk, meat, eggs, and fats. Their persistence stems from the presence of multiple, highly stable carbon–fluorine bonds, which render them resistant to degradation. PFASs tend to bioaccumulate and can be transported over long distances in the environment, leading to accumulation within the food chain. Furthermore, unlike many toxic pollutants that are mainly concentrated in lipids, PFASs exhibit a strong affinity for proteins and matrices with multiple physicochemical properties [99,104,105].

Derivatives of PAHs and HAAs, thermogenic compounds which are formed de novo during the thermal processing, smoking, drying, or roasting of protein-rich foods (such as meat and fish) at high temperatures, typically between 100 °C and 300 °C, are most abundant in meat, fish, and smoked meats [85,89,90,91,93,104,105,106,107,108,109,110,111].

### 3.3. Processed and Mixed Matrices

Processed and combined matrices, formed of a mixture of plant and animal-derived ingredients, represent a significant analytical challenge due to their high heterogeneity and the extensive processing they undergo. Intensive thermal and industrial processing facilitates the retention and bioaccumulation of environmental contaminants, as well as the de novo formation of toxic compounds, including HAAs and PAHs derivatives, from endogenous precursors under high-temperature conditions. Furthermore, the recent literature highlights the presence of other pollutants, such as PFASs and pesticides, in processed foods like fried potatoes, and reports on pastries and bread in which high levels of HAAs and PAHs were determined [89]. Other examples in processed matrices include cereal-based (multigrain) baby products. Research has shown the persistent presence of alkaloids in these samples, as well as the need to quantify mycotoxins in cereal raw materials (such as rice and oats) used in their manufacture [81,92]. Patulin, on the other hand, is a known problem in apple-derived products that have been processed or are intended to be consumed by children [95].

All compounds have an affinity for specific matrix components, including proteins and lipids, which enables their presence in foods with high concentrations of these macronutrients [77,90,91,92,93,97,98,99,109,110,111].

**Figure 1:**
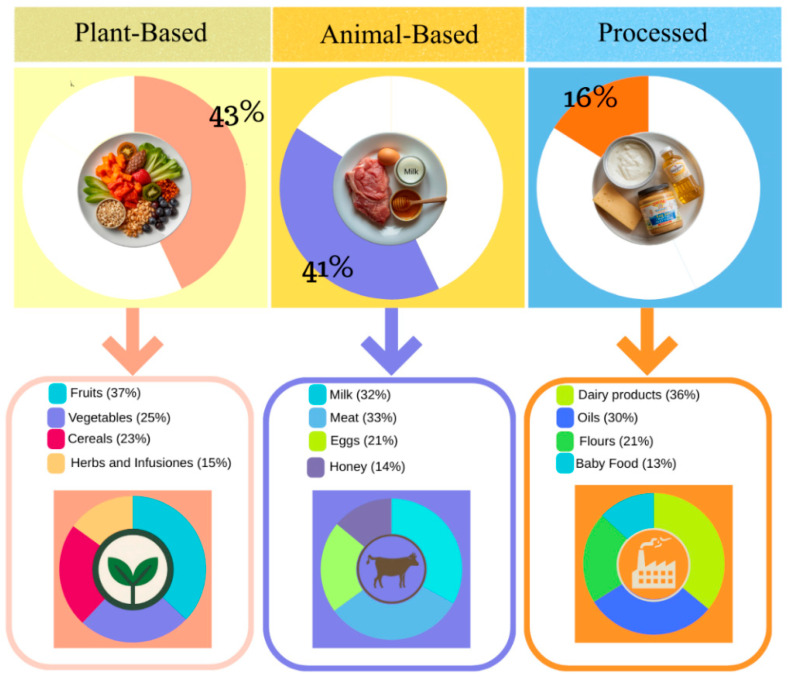
Distribution of research reporting major classes of exogenous toxic contaminants across plant-based, animal-based, and processed food categories (2020–2025).

In summary, Figure 1 provides a comparative overview of the most frequently investigated exogenous toxic compounds across major food categories based on surveillance studies published between 2020 and 2025. Rather than reflecting absolute contamination levels, the distributions shown highlight analytical and regulatory priorities, as the compounds most often reported correspond to those subjected to the most stringent MRLs established by international regulatory authorities. The prominence of specific compound–matrix pairings in the recent literature underscores areas where compliance with MRLs remains analytically challenging and where continued methodological advances are essential for effective food safety surveillance.

## 4. Sample Preparation Techniques Exogenous Toxic Compounds in Food Analysis

**Figure 2:**
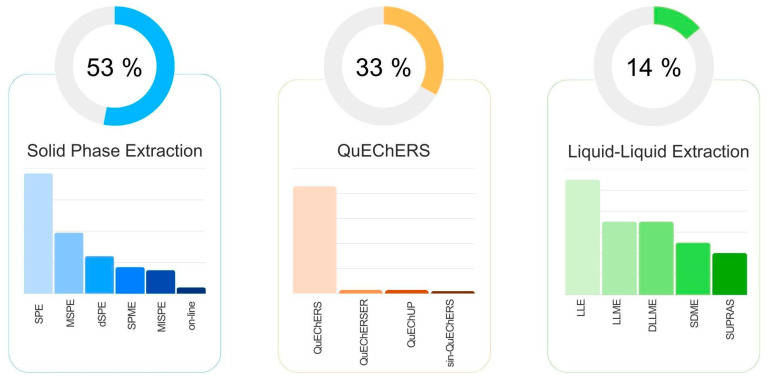
Frequency of sample preparation methods used for detecting exogenous toxic compounds in food samples by LC-MS/MS.

The sample preparation process, particularly the extraction step, is crucial for the removal of matrix interferences, as well as the isolation and concentration of analytes to ensure high-quality measurements [112,113]. Various techniques are available for sample preparation mediated by a sorbent or a solvent. A survey of literature published between 2020 and 2025 regarding the determination of exogenous toxic compounds in food analysis by LC-MS/MS, as indexed in Scopus, shows (Figure 2) that solid-phase extraction is the predominant technique, applied in approximately 53% of the reviewed publications. QuEChERS is utilized in 33% of cases, while liquid–liquid extraction is performed in only 14% of the studies examined.

### 4.1. Solid-Phase Extraction

Solid-phase extraction is undoubtedly the most used technique for the treatment of food samples due to its ability to modulate selectivity by using specific sorbent materials. Conventional SPE methods are typically conducted using cartridges or well plates packed with solid adsorbents. The extraction procedure involves four key steps: conditioning, sample loading, washing, and elution [113]. While these methods remain widely used for processing complex matrices due to their robustness and reliability, recent advancements in analytical chemistry have driven the development of innovative approaches aimed at improving the analysis of complex food samples.

In this regard, magnetic solid-phase extraction (MSPE) is an advanced sample preparation technique that combines the benefits of conventional SPE with the simplicity of magnetic separation, eliminating the need for complex, packed columns or time-consuming filtration and centrifugation steps [114]. The efficiency and selectivity of MSPE are directly influenced by the properties of the adsorbents, which are typically composed of magnetic nanoparticles (MNPs) functionalized with a specific material, such as carbon-based materials, metal–organic frameworks (MOFs), covalent organic frameworks (COFs), and molecularly imprinted polymers (MIPs) [115]. In food analysis by LC-MS/MS, magnetic COFs (MCOFs) have garnered significant attention due to their exceptional properties; although these materials offer a functional versatility that transcends the specific analytical applications discussed here, they are utilized in this work as specialized sorbents for efficient sample preparation. These are new types of porous crystalline materials consisting of light elements (C, B, O, Si, N) bonded by strong covalent bonds. Equipped with a customizable platform, these materials are designed to meet the growing demand for high performance and environmental sustainability. This approach is especially advantageous for complex analyses involving a variety of exogenous analytes across multiple food matrices, where achieving high recovery rates and low detection limits is essential to maintaining food safety standards. Several studies show the efficient application of MCOFs for the extraction and analysis of antibiotics (especially sulfonamides, fluoroquinolones, and macrolides) with recoveries from 80% to 110% and low detection limits (LODs) and quantification limits (LOQs), ranging from 0.1 to 2 μg kg^−1^ [116,117,118,119,120]. Another interesting application of MCOFs is the one reported by Wang et al. [121] for the determination of heterocyclic aromatic amines in cake using a novel magnetic adsorbent that introduced carboxylic groups, providing additional binding sites. This sorbent demonstrated excellent conditions, providing an accurate tool for HAAs detection. As shown in Table 1, most food matrices analyzed using MSPE include animal-origin foods such as milk [120,122,123,124,125,126], meat [93,116,127,128], eggs [117,119,123], honey [118,129], and processed food [121].

Although MSPE presents significant advantages in terms of selectivity, detection and quantification limits and multi-analyte applicability, the variability of magnetic materials and the need for specialized synthesis of sorbents for each application, limits the widespread use of the technique in the field of food security analysis.

In the same way that MSPE, dispersive solid-phase extraction (dSPE) represents an attractive alternative for the cleanup and preconcentration of analytes from complex matrices. Relying on direct contact between the sorbent and the sample matrix, leading to an extremely rapid extraction process and making it particularly well-suited for high-throughput applications. For example, a novel extraction sorbent composite of MWCNTs/NH2-MIL-101(Fe) was synthesized by Huang et al. [133] to extract 6 kinds of neonicotinoid pesticides in food products and determined with UHPLC-MS/MS. The proposed method achieved a low LOD (0.01–0.07 μg kg^−1^) and high extraction recoveries (78–101%). Other analytes have also been detected in various food matrices using dSPE (Table 1). Mycotoxins in vegetable oils [132], antibiotics and veterinary drugs in eggs [130,131], PFASs in maize and wheat [157] and polycyclic PAHs in seafood [158] are some of the most relevant.

While magnetic solid-phase extraction and dispersive solid-phase extraction offer significant advantages in sample preparation—such as achieving low detection limits and high recoveries—sometimes alternative strategies are needed due to the complexity of certain food matrices. In these cases, the development and application of highly selective materials become essential to address more demanding analytical requirements, even in the presence of challenging matrix interferences. In this sense, although C18 appears as the most widely used solid phase in the research works reviewed, there has been a clear rise in using molecularly imprinted polymers for solid-phase extraction. MIPs are a type of polymer that is intentionally engineered to recognize a specific target molecule or group of compounds with high selectivity. These features make them extremely versatile and effective in various analytical applications in food analysis. As shown in Table 1, most frequent applications were mainly used for the detection of antibiotics and veterinary drugs in milk [114,134,159].

Within the ongoing advancement of analytical chemistry, micro-solid-phase extraction (µSPE) has also appeared as a new approach for the clean-up and preconcentration strategy in recent years [113,160]. This technique is based on dispersing a microgram-level amount of sorbent into the solution containing the analyte. Compared with conventional SPE methods, a larger surface area is obtained, enabling quicker equilibration and enhancing the equilibrium ratio. As a result, it delivers improved extraction efficiency and reduces the overall sample preparation time. The simplest format used is pipette-tip μSPE (PT-μSPE), in which the sorbent is placed in a tip, and extraction is handled using a pipette. One interesting application is the one used by Ning et al. [136] in which a PT-μSPE technique was applied to extract sulfonamide residues in food samples before LC-MS/MS determination. In addition, Behbahan et al. [135] reported the preparation of modified biochar and its applications in PT-µSPE for the extraction of twelve pesticides from fruits and vegetables. Table 1 shows that both approaches yielded analytical results consistent with those obtained using commercial adsorbents, indicating acceptable performance.

Beyond these manual or semi-automated microextraction techniques, the pursuit of enhanced efficiency and throughput has led to further innovation. In recent years, there has been a noticeable increase in the number of publications dedicated to the development of automated and online SPE methodologies [160]. An example of fully automated online SPE configuration is that applied for Sun et al. [137] for the determination of PFASs in seafood using an ionic covalent organic framework (TPB-BFBIm-iCOF) as an SPE sorbent. This approach offers lower detection limits and better recovery than existing methods (Table 1), suggesting its value for PFAS monitoring in food samples.

The inherent versatility and efficiency of solid-phase extraction establish it as the most relevant technique in food analysis by LC-MS/MS. Furthermore, the implementation of eco-friendly sorbents and assisted techniques improve the sustainability of the process and enhance the performance of the technique to satisfy the principles of green chemistry. This translates into the achievement of high enrichment factors, coupled with a notable reduction in the use of hazardous reagents, lower operational costs, and optimized analysis times.

### 4.2. QuEChERS

Since its introduction in 2003 [161], QuEChERS has become one of the extraction methodologies that better aligns the requirements of safety and sustainability with high analytical performance. The method involves three steps: extraction, clean-up, and detection. In the first extraction step, water and an organic solvent are added to the homogenized sample, obtaining phase separation through the salting-out effect due to the addition of inorganic salts (NaCl and MgSO_4_). The second step of the procedure is dSPE, which is performed to remove the interfering compounds co-extracted with analytes in the first step. Finally, the instrumental analysis is typically performed using chromatography and mass spectrometry. In the beginning, QuEChERS was focused on pesticide residue detection in agricultural products and then evolved into the standard preparation method for pesticide residue analysis in the USA and the European Union.

For the reasons mentioned above, in the review of published research works on QuEChERS, the applications for pesticides in samples of plant origin stand out (Table 1). A representative example is reported by Schiano et al. [141], in which seven pesticide residues in extra virgin olive oil were determined by LC-MS/MS. The extraction step was made using in-house analytical procedure based on QuEChERS consisting of an extraction with 10 mL of acetonitrile (ACN) followed by a clean-up step using C18/PSA sorbents. Under optimal conditions, all the pesticides showed recovery between 70 and 120% and RSD <20%. Similarly, Prata et al. [145] reported the monitoring of twenty-one pesticides and four aflatoxins in baby food products by QuEChERS extraction combined with dSPE. Table 1 presents the use of traditional QuEChERS methodology for analyzing pesticides [138,139,140] and mycotoxins [142,143,144] across various food matrices. This approach yields low limits of detection and limits of quantification, as well as high recovery rates suitable for multi-analyte analysis.

Several other attempts have been made to expand its applicability to more complex matrices and a broader range of contaminants. In this context, some variations have been reported with the aim of analyzing multiclass compounds with varying polarity, such as QuEChERSER [146,162,163] and QuEChUP (Quick, Easy, Cheap, and Ultra-high Productivity) [147,164]. Furthermore, a few publications have reported on how traditional QuEChERS methods have been modified to become more environmentally friendly. The Sin-QuEChERS method was used for non-targeted high-throughput rapid screening and quantitative analysis of residual pesticides and metabolites in green teas [148]. The sample was extracted with 0.1% formic acid in ACN and purified with a nano-solid-phase extraction column. The results presented in Table 1 for this miniaturized method indicate strong linearity, low limits of quantification, and high recovery rates. These findings suggest that the methodology is suitable for quantitative analysis of potential residual pesticides in tea samples. In addition, a modified QuEChERS method was developed by Zhou et al. [149] to identify multiclass pesticide and veterinary residues in aquatic products using chitosan microspheres as the cleanup sorbent. Compared to conventional synthetic sorbents, this eco-friendly sorbent showed good purification performance (Table 1) and achieved recoveries ranging from 64% to 116% and low LOD and LOQ (0.5–1.0 and 1.0–2.0 μg kg^−1^, respectively).

Undoubtedly, QuEChERS offers excellent results for hundreds of different compounds in many food matrices, but this method also encounters certain limitations and challenges in specific applications leading to future innovations.

### 4.3. Liquid–Liquid Extraction

Liquid–liquid extractions involve the partitioning of an analyte between two immiscible liquids, typically aqueous and organic phases, based on the analyte’s solubility in the chosen extraction solvent. Common solvents for conventional LLE include 1-octanol, hexane, chloroform, carbon tetrachloride, toluene, ethyl acetate, and diethyl ether; however, their application is discouraged due to concerns regarding toxicity and environmental impact [165]. Over the last decade, advances in food analysis have focused on improving efficiency, reducing environmental impact, and minimizing costs through the development of miniaturized techniques and greener solvents [166]. In this sense, several techniques have been developed, including liquid-phase microextraction (LPME), which is one of the most frequently used applications employed for the analysis of exogenous toxic organic compounds in food analysis. The main configurations of LPME applied (Figure 2) were dispersive liquid–liquid microextraction (DLLME) and single-drop microextraction (SDME).

DLLME operates by generating a dispersion between a water-insoluble solvent and the aqueous sample. The large surface area achieved through this dispersion enables efficient extraction within short timeframes. Various methods can be employed to facilitate dispersion; the chosen approach depends not only on the characteristics of the sample but also on the analyst’s preference [166]. For example, Jai et al. [150] developed a DLLME procedure to extract 15 mycotoxins from green tea using two combinations of dispersion and extraction solvents: acetonitrile/ethyl acetate was used in the first step, and methanol/chloroform was used in the second step of the extraction. The results showed recoveries between 81 and 125% and RSD < 16%. This method was faster and more cost-effective than current SPE procedures.

In SDME, extraction is performed using a drop suspended from the tip of a micro-syringe needle without the need for specialized equipment, making the process straightforward and cost-effective. After extraction, the drop is withdrawn and placed directly into the analytical instrument [166]. Bochetto et al. [153] provided an example of this sample preparation method for the analysis of mycotoxins in amaranth seeds. The methodology described applied in-phase liquid–liquid microextraction based on the solidification of a floating organic drop, followed by a double solvent-assisted back-extraction technique. Compared with conventional techniques, this approach used lower volumes of solvent and resulted in lower LOD and LOQ values, high recoveries, and RSD% < 10%. Further LLME applications are listed in Table 1.

Other relevant liquid-phase configurations, such as hollow fiber–liquid phase microextraction (HF-LPME), have not been widely applied for analyzing exogenous toxic compounds in food samples using LC-MS/MS. This is likely due to issues like clogging susceptibility and variability in sample matrices. Although HF-LPME has seen limited adoption in complex food sample preparation, it remains valuable for biological and environmental samples that involve more homogeneous matrices [7].

As previously discussed, advances in LLE have been driven by the need to improve efficiency, reduce environmental impact, and minimize costs, especially in the analysis of exogenous toxic compounds in food. Building on this trend, the review of scientific publications regarding liquid–liquid extraction in food analysis by LC-MS/MS shows an increased application of natural solvents. This shift reflects ongoing efforts to develop greener sample preparation methods that address the toxicity and environmental concerns associated with traditional organic solvents, further enhancing the sustainability of modern food analysis. Deep eutectic solvents (DESs), natural DESs (NADESs), ionic liquids (ILs) and supramolecular solvents (SUPRASs) are among those being used more frequently, contributing to research into more efficient and environmentally sustainable processes.

Deep eutectic solvents are formed by non-covalent interactions between hydrogen bond donors and acceptors. They feature low toxicity, are biodegradable, have adjustable properties, and maintain high solubility in mild conditions. DESs have proven effective for extracting bioactive compounds, making them useful tools for detecting and analyzing substances within food matrices [167]. For example, Table 1 summarizes the work of Del Bosco et al. [151], who employed hydrophobic eutectic solvents to extract 19 pesticides from wine. In addition, Abdallah obtained high recovery rates for three pesticides from fruit by implementing a novel approach that integrated hydrophobic deep eutectic solvent with vortex-assisted liquid–liquid microextraction (VA-LLME) [155].

On the other hand, when DESs are synthesized with natural molecules—choline chloride, organic acids, sugars, and amino acids—NADESs are obtained. Like DESs, NADESs possess tunable solubility in organic solvents, low vapor pressures, high conductivity, and thermal stability. They generally exhibit lower toxicity, and most organisms can metabolize NADESs, contributing to their biocompatibility. They are also suitable for reuse and recycling [7]. As can be seen in Table 1, NADESs were employed for LLME in the analysis of mycotoxins in olive oil by Pradanas-Gonzales et al. [84], as well as for DLLME-SFO in apple-based products by Carbonell-Rozas et al. [154]. In addition, terpene derivative-based NADESs served as an extraction solvent in DLLME for studying multiclass metabolites of pesticides and PAHs in food products of animal origin [152]. The established method demonstrated low LOD and LOQ with recoveries between 81% and 112% (Table 1).

Ionic liquids are salts that contain organic cations—imidazolium, pyridinium, pyrrolidinium—and inorganic anions like chloride and dicyanamide. As green solvents, some of their unique properties include negligible vapor pressure at a wide range of temperatures, high thermal stability, and high viscosity [7]. Their tunable physicochemical properties make them an attractive alternative to traditional organic extraction solvents. Several studies have reported the use of ionic liquids as dispersant solvents for solid-phase extraction of antibiotics from milk [168,169,170].

Finally, SUPRASs are another promising alternative to organic solvents in sample preparation because their tunable microenvironments and multiple binding sites allow diverse analyte interactions. These nanostructured liquids form through self-assembly and coacervation in colloidal solutions of water-immiscible amphiphilic compounds, such as surfactants or long-chain carboxylic acids. Muñiz-Bustamante et al. [156] employed SUPRASs for mycotoxin detection in food, as detailed in Table 1. Compared to conventional approaches—which frequently require matrix-specific protocols, substantial quantities of solvents, and costly consumables—the SUPRAS method presents a more streamlined, sustainable, and cost-effective alternative. Nonetheless, ongoing enhancements are necessary to further strengthen the analytical reliability of SUPRAS-based techniques.

In summary, despite the advancements made in the field of sample preparation, each technique presents certain drawbacks, such as limited applicability to highly complex matrices, variable recoveries depending on analyte and sample type, and issues related to solvent toxicity and waste generation. A comparative overview of the main food matrices investigated, the exogenous toxic compounds commonly reported in each matrix, the associated analytical challenges, and the principal extraction techniques employed is provided as complementary information in Table S2 (related references [37,38,39,40,41,42,43,44,45,46,47,48,49,50,51,52,53,54,55,56,57,58,59,60,61,62,63,64,65,66,67,68,69,70] are cited in the Supplementary Materials). This table highlights the strong influence of matrix composition on extraction efficiency, matrix effects, and method selectivity, underlining the need for tailored sample preparation strategies when developing multiresidue analytical methods for complex food systems. Ongoing research aims to refine these methods by introducing innovative extraction materials, optimizing solvent selection, and integrating automation to further enhance precision and sustainability, thereby enabling sensitive LC–MS determination of exogenous toxic compounds across diverse food matrices. As the field continues to evolve, the adoption of greener materials and miniaturized extraction systems is expected to play a pivotal role in shaping future sample preparation strategies for food analysis.

## 5. Integration of Advanced Sample Preparation with UHPLC–MS and Spectral Databases

The effectiveness of the environmentally friendly extraction techniques described in the preceding section is fundamentally dependent on the performance of the instrumental detection phase. In food analysis, understanding matrix-driven interferences is particularly relevant when dealing with plant, animal, and processed products, as their complex composition directly determines both extraction efficiency and analyte recovery [74,75,76,77,78]. While Section 4 highlighted sustainable methods for isolating contaminants, powerful instrumentation cannot fully compensate for inadequate sample preparation; therefore, the efficiency of the cleanup steps remains a critical determinant of the final analytical performance.

### 5.1. Instrumental Requirements for Diverse Food Extracts

Methodological advancements have been driven by the integration of chromatographic separations with increasingly sensitive tandem mass spectrometry and high-resolution mass spectrometry (HRMS) detectors. Together, these components enable the multiresidue, multi-matrix workflows described in this review to achieve detection limits in the low ng kg^−1^ to µg kg^−1^ range for priority exogenous contaminants [1,2,3,4]. Additionally, the concurrent development of ion mobility spectrometry (IMS) coupled to UHPLC-HRMS provides an orthogonal separation dimension, improving the discrimination of isobaric and isomeric species in complex food matrices [14].

A major conceptual shift has been the integration of contaminant analysis with foodomics, where metabolite profiling, biomarker discovery, and exposure assessment strategies increasingly overlap with analytical chemistry and toxicology [4,15]. Consequently, contemporary UHPLC-MS workflows contribute not only to compliance monitoring but also to a deeper understanding of dietary exposure and its potential health risks.

From a chromatographic standpoint, the diversity of contaminants requires specialized stationary phases to manage the extracts provided by SPE or QuEChERS. Alternative phases, such as HSS T3 or phenyl-hexyl, have improved the retention of polar and aromatic analytes, while modern C18 columns packed with superficially porous particles allow for fast gradients and high peak capacity [1,12,13].

### 5.2. Linking Sustainable Extraction to Green Instrumental Workflow

The shift toward green analytical chemistry (GAC) and Green Sample Preparation (GSP) detailed in Section 4 extends directly into the instrumental workflow [6,8,9,10]. Efforts now focus on reducing solvent consumption and minimizing hazardous reagents across the entire process. Parallel to the miniaturization of extraction formats, miniaturized LC systems have become increasingly viable. Microflow UHPLC (10–50 µL min^−1^) improves electrospray ionization efficiency, reduces solvent consumption by an order of magnitude, and often decreases the matrix effects caused by residual co-extracted components [6,8,10,11]. The sustainability of these integrated workflows can now be evaluated using standardized metrics like AGREE and AGREEprep, ensuring that the green benefits gained during extraction are not lost during analysis [9,10].

### 5.3. High-Resolution Screening and Spectral Identification

The introduction of HRMS platforms—primarily ORBITRAP and QTOF—has expanded the analytical scope beyond the regulated analytes targeted by conventional extraction toward suspect and non-target screening [7,14,15]. These systems permit retrospective data mining, allowing for the discovery of emerging contaminants or transformation products within the dense full-scan datasets generated by the eco-friendly extracts. Furthermore, and as mentioned, the development of IMS provides an orthogonal separation dimension, improving the discrimination of isobaric and isomeric species that may co-elute in complex food matrices [7,14,15,171].

Automation and digitalization are also transforming the field. Online sample-preparation systems directly hyphenated to UHPLC–MS minimize manual handling and reduce errors [11,16]. Machine learning tools now assist in chromatographic optimization, retention-time prediction, and HRMS data interpretation [7,171], accelerating data processing and facilitating rapid quality-control decisions. Persistent challenges include matrix complexity, ion suppression, carry-over, and the lack of standardized non-targeted workflows [3,5,12]. Additionally, the still limited availability of food-oriented spectral libraries restricts the routine implementation of HRMS [7,14,15].

Ongoing efforts towards integration of miniaturized LC platforms, HRMS detection, and chemometric or in silico tools are expected to define the next generation of analytical workflows. Emphasizing green analytical chemistry principles and harmonized validation metrics will guide future innovations in food contaminant analysis [8,9,10,13].

Identification of organic contaminants in foods is becoming increasingly reliant on high-quality spectral libraries and curated chemical databases. These resources have expanded substantially, supporting both targeted monitoring and broader exploratory screening [172,173,174,175]. For routine surveillance, laboratories rely heavily on experimentally verified MS/MS spectra available in vendor-specific libraries. These collections provide accurate fragmentation patterns and, in many cases, retention time information for pesticides, veterinary drugs, mycotoxins, food-contact chemicals, and heat-induced contaminants. Additionally, many institutions develop internal spectral libraries to stabilize retention times and reflect laboratory-specific UHPLC conditions [174,176]. On the other hand, for broader exploratory work, public repositories such as MassBank, MoNA, GNPS, HMDB, and mzCloud provide thousands of experimentally collected spectra. These resources include metadata, such as collision energies, isotope patterns and, in some cases, retention indexes, that improve match reliability. In complex food matrices, analysts simultaneously consider spectral similarity, mass accuracy, isotope-pattern fit, and retention constraints to avoid misidentifications [172,174]. Suspect-screening strategies depend on specialized chemical metadata databases for compounds without experimental MS/MS spectra. These databases include the NORMAN Suspect List Exchange, EPA CompTox, PubChem Lite, and EFSA-related lists, which offer identifiers (InChIKeys, SMILES), exact masses, predicted fragments, and toxicity annotations [173,175,177]. Such resources are essential for identifying emerging contaminants, transformation products, and food-contact substances in HRMS workflows. When experimental spectra are unavailable, in silico fragmentation tools such as SIRIUS/CSI:FingerID, MetFrag, MS-FINDER, and CFM-ID are commonly used. These tools are being more frequently integrated with retention time prediction models and ion-mobility collision cross-section (CCS) values. The growing availability of both measured and predicted CCS databases enhances the discrimination of closely related or isomeric contaminants [176,178]. QA/QC practices, including the adoption of the Schymanski confidence-level framework, detailed reporting of software tools, spectral libraries, and ranking criteria, substantially improve the reliability of contaminant identification [172,178]. Interlaboratory comparisons highlight that reproducibility significantly improves when preprocessing parameters and scoring strategies are standardized [175]. Overall, the growth of spectral repositories, suspect lists, and in silico tools has transformed UHPLC–MS-based contaminant analysis. Laboratories can now perform broader surveillance, identifying both regulated substances and newly emerging contaminants. These advancements ultimately strengthen food safety monitoring and early-warning systems [172,173,174,175,176,177,178,179,180].

## 6. Green Metrics in Food Analysis

Sample preparation is a critical determinant of analytical performance. Accordingly, assessing the sustainability of LC–MS methodologies is essential, as this widely used approach to food analysis entails substantial consumption of solvents, energy, and materials, which must be managed responsibly to minimize the environmental footprint of analytical laboratories.

In this regard, the concept of GAC has been established in the literature for more than two decades. GAC represents a scientific paradigm that integrates sustainability principles into analytical laboratories and industries. The GAC is grounded in the twelve principles of green chemistry, which provide a comprehensive framework for developing environmentally friendly methodologies [181]. Meanwhile, the ten principles of Green Sample Preparation [182] serve as a guide for establishing the roadmap toward the development of overall greener analytical workflows. These principles emphasize the use of safe solvents and reagents, renewable, recycled, and greener alternatives solvents (e.g., NADES, DES and IL). Reusable materials minimize waste volume generation and energy consumption. They also promote high sample throughput, miniaturization (microextraction techniques of LLE or SPE), procedure simplification or automation, and the assurance of operator safety [9]. Moreover, the application of green metrics to assess the greenness of sample preparation methods is highlighted, underscoring the contribution of GSP to the overarching goals of sustainability. Importantly, green sample preparation is not a new subdiscipline, but a principle that fosters sustainable methodological development through the adoption of environmentally friendly sample preparation strategies.

Another important concept is White Analytical Chemistry (WAC), introduced by Nowak et al. [183]. This approach complements GAC by extending its principles beyond environmental aspects to include analytical efficiency and practical functionality. While GAC focuses mainly on ecological aspects, WAC proposes integrating environmental, analytical, and practical criteria. Traditionally, the development of analytical procedures has been guided by performance parameters such as the limits of detection (LOD) and quantification (LOQ), recovery, precision, and linear range. In addition to these analytical criteria, ecological considerations and cost-related aspects must also be considered. Therefore, the selection of an analytical method should evaluate all these dimensions in an integrated manner.

Since 2002, numerous green metrics have been developed to assess the greenness and environmental performance of analytical procedures, reflecting the growing interest in sustainable analytical practices [9]. An overview of the evolution of the GAC metrics listed above is provided in Table 2. Whether applied individually or in combination, these metrics offer a robust framework for evaluating the environmental impact, analytical performance, innovation and/or practical applicability of analytical procedures.

According to Lei Yin et al. [13], some metrics are developed for specific analytical applications, e.g., AGREEmip, a quantitative tool designed to assess the environmental impact of biopolymer synthesis, whereas other metrics are universally applicable across most analytical methods (AGREE, AGREEprep, GAPI, and RGB with its complementary version). The evolution of metrics ultimately paved the way for comprehensive frameworks (GAC and WAC), which aim to balance environmental, analytical, and practical aspects of the development and assessment of analytical procedures. As noted by several authors, each metric presents its own advantages and limitations [13,211,212].

**Figure 3:**
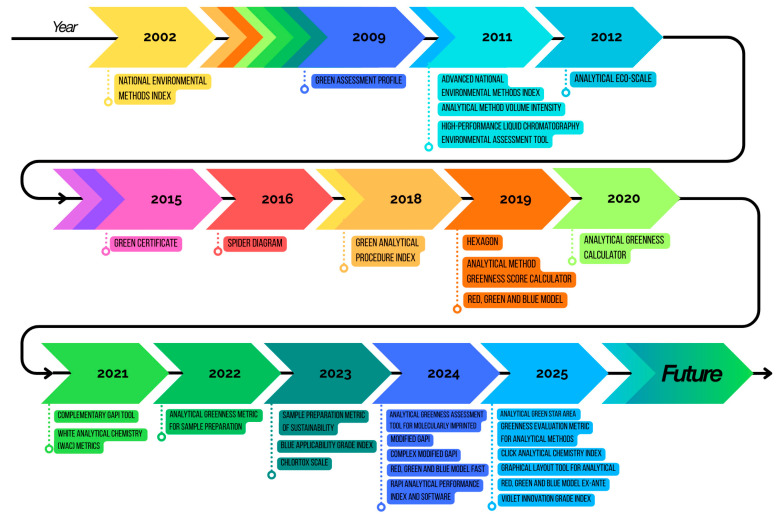
Timeline of the development of green analytical metrics from 2002 to the present, highlighting the key milestones and methodological advances in the evaluation of analytical greenness.

As illustrated in Figure 3 the development of green analytical metrics has increased steadily over time, reflecting the growing interest in sustainable practices within analytical chemistry. Notably, more than 60% of all metrics reported in the literature have been published within the past five years, highlighting a recent surge in research focused on evaluating the environmental impact, analytical performance, and practical applicability of analytical procedures. This trend underscores the growing importance of establishing standardized tools to assess and promote greener analytical methodologies. Furthermore, these metrics continue to evolve, improving both in software-based implementations and in their capacity to balance environmental, analytical, and practical considerations.

**Figure 4:**
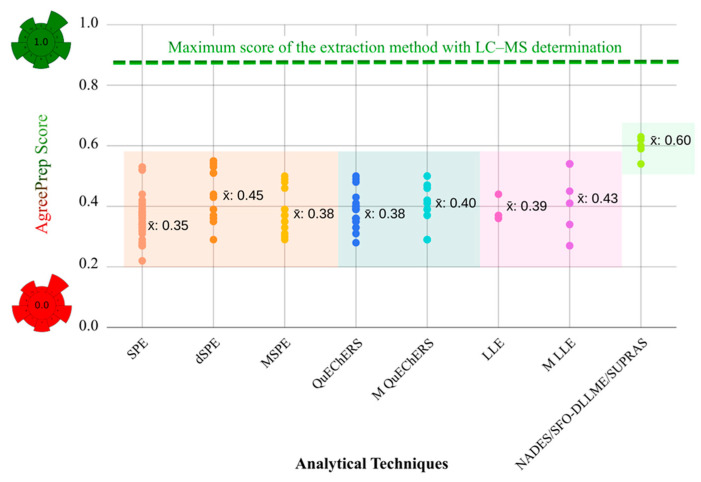
AGREEPrep scores and distribution of the extraction techniques evaluated (n = 108) for the determination of mycotoxins, veterinary drugs, antibiotics, non-polar pesticides, heterocyclic aromatic amines, polycyclic aromatic hydrocarbons, and polyfluoroalkyl substances by liquid chromatography coupled with mass spectrometry. The techniques included dispersive solid-phase extraction (dSPE), solid-phase extraction (SPE), magnetic solid-phase extraction (MSPE), Quick, Easy, Cheap, Effective, Rugged and Safe (QuEChERS), Unconventional QuEChERS (M QuEChERS), Liquid–Liquid Extraction (LLE), and Unconventional Liquid–Liquid Extraction (M LLE), supramolecular solvents (SUPRASs), Natural Deep Eutectic Solvents (NADES), and Dispersive Liquid–Liquid Microextraction coupled with a Floating Organic Drop (SFO-DLLME).

As discussed in Section 4, determination of exogenous toxic organic compounds in foods at trace concentrations generally involves four main steps: sampling, sample preparation, instrumental analysis, and data processing. The sample preparation is considered the most critical stage, as it largely determines the accuracy, recovery, and reliability of the analytical results [7]. In recent decades, alternatives to conventional organic solvents have gained increasing importance in sample preparation, as they offer the potential to minimize or eliminate the toxicity, volatility, and flammability associated with traditional solvents. The introduction of NADESs, DESs, ILs, and SUPRASs has provided greener, safer, and more efficient alternatives for the sample preparation stage. The implementation of these new solvents aligns with the principles of GAC. In this context, when green solvents are applied in extraction techniques, green metrics yield higher scores, indicating greener methods compared to traditional methodologies that rely on conventional solvents and reagents. This is consistent with their lower environmental impact and reduced operator risk. Furthermore, microextraction techniques employing green solvents achieve even higher green metric scores, as they require significantly smaller solvent volumes, generate less waste, and consequently enhance the overall greenness of the analytical method (Figure 4).

In summary, the integration of the use of green solvents, microextraction techniques, and assessment metrics represents significant progress toward more sustainable analytical procedures. These developments not only reduce the environmental and health impacts associated with traditional methodologies but also promote efficiency, innovation, and reliability in modern analytical science.

### Overview of Extraction Techniques Assessed with the AGREEprep Metric

A Scopus search conducted for this review indicates that fewer than 30 studies published over the past five years have applied green metrics to LC–MS-based determinations of exogenous toxic organic compounds in food matrices. This number is markedly lower than expected considering the extensive body of published LC–MS workflows and is also lower than that reported by Lei Yin et al. [13], highlighting the limited adoption of greenness assessment in this area of analytical research. In addition, AGREEprep was applied to evaluate the greenness of extraction techniques used for the LC–MS determination of exogenous toxic compounds in food matrices. AGREEprep is a user-friendly, freely available tool designed to comprehensively assess the greenness of sample preparation procedures based on the ten principles of green sample preparation [13]. Other greenness assessment metrics, such as GAPI and its variants, were not considered due to the limited availability of the required methodological data in the reviewed publications. A Scopus search was performed to identify scientific articles published between 2020 and 2025 corresponding to each analyte class included in this review. From the retrieved records, DOIs were collected, and three publications per analyte per year were randomly selected using ChatGPT (OpenAI), GPT-5.2 version. As only a limited number of studies applied more than two sample preparation approaches, a total of 108 extraction procedures (n = 108) were evaluated. Based on the sample preparation strategies reported for different food matrices, the methods were classified as follows: dispersive solid-phase extraction (n = 12), solid-phase extraction (n = 33), magnetic solid-phase extraction (n = 15), Quick, Easy, Cheap, Effective, Rugged, And Safe (n = 23), modified QuEChERS (n = 9), liquid–liquid extraction (n = 3), modified liquid–liquid extraction (n = 8), supramolecular solvents (n = 1), natural deep eutectic solvents (n = 1), and dispersive liquid–liquid microextraction with a floating organic drop (n = 3). Due to their infrequent occurrence, SUPRASs, NADESs, and SFO-DLLME were grouped into a single category (Figure 4). The randomly selected techniques share four fixed AGREEprep criteria:

- Criterion 1—“Sample preparation placement” was identical across all methods, as sample pretreatment was performed ex situ in the laboratory.
- Criterion 3—“Target sustainable, reusable, and renewable materials” was uniformly scored, since the materials used were neither sustainable nor renewable, although they were reused multiple times.
- Criterion 6—“Maximize sample throughput” was benchmarked against solid-phase extraction (SPE) as the reference technique, using a 24-position vacuum manifold system that enables parallel sample processing.
- Criterion 9—“Post-sample preparation configuration for analysis” was constant for all approaches, as LC–MS was employed in every case.

Under the default AGREEprep weighting scheme, the application of these four fixed criteria resulted in an identical initial baseline score of 0.85 for all evaluated techniques. This score of 0.85, instead of 1, accounts for the “fixed costs” of the detection phase before assessing the specific extraction technique. This is because certain fixed parameters—Instrumental Energy and Solvents (GSP Principle 9) and Laboratory Placement (GSP Principle 1)—are shared across the entire dataset.

As shown in Figure 4, conventional techniques (SPE, LLE, and QuEChERS) and their modified variants exhibit comparable average scores, ranging from 0.35 to 0.45. This trend is primarily attributed to high solvent and reagent consumption, leading to increased waste generation, the predominant use of solvents such as acetonitrile, methanol, and formic acid—which increases the number of hazard pictograms in the assessment—and the relatively high number of procedural steps involved (typically ≥4 per method). In addition, the average sample mass used was 5 g. These variables result in scores that negatively affect the greenness of the techniques. The QuEChERS approach is the least time- and energy-intensive among the evaluated procedures [160]. However, the volumes and types of solvents employed are comparable to those used in other analytical techniques, including SPE, LLE, and their modified variants. As summarized in Table 1, all these methods are compatible with mass spectrometric detection, enabling accurate identification, quantification, and confirmatory analysis of target analytes [213].

On the other hand, the SUPRAS, NADES, and SFO-DLLME-based approaches show a substantial increase in greenness (Figure 4) when microextraction strategies and green solvents are employed, mainly due to reduced solvent consumption, minimal waste generation, and inherently safer chemical properties. Among these, SUPRAS exhibits performance comparable to NADES, generating low waste and requiring smaller amounts of solvents and reagents [160], while also providing green attributes for the sample preparation process and enhancing extraction efficiency [211]. Nevertheless, although metrics have been developed for specific analytical applications (e.g., AGREEmip), no dedicated tool is currently available to evaluate the greenness of the synthesis of NADES, ionic liquids, deep eutectic solvents, or framework-based materials such as metal–organic and covalent organic frameworks, nor to account for their use in sample preparation in line with the principles of green analytical chemistry and green sample preparation.

In this context, Souza Futigami, L. et al. [158] compared the greenness of a method employing natural solid sorbents for SPE in the determination of PAHs in seafood with a standardized procedure described in the *Manual of Analytical Quality Assurance* (MAPA). Using the AGREEprep metric, the standardized method achieved a score of 0.04, whereas the proposed approach reached 0.52. These findings indicate that compliance with regulatory standards does not necessarily translate into a favorable environmental performance. Accordingly, greenness assessment can reveal trade-offs in analytical procedures, whereby a method may offer high accuracy yet impose a substantial environmental burden, or alternatively exhibit improved sustainability but limited sensitivity, as is often observed for screening-based mycotoxin analyses [213].

In summary, the application of green metrics in food analysis using LC-MS techniques provides a solid approach for assessing the sustainability of analytical procedures. The development of green metrics, based on GAC and GSP principles, enables a more objective evaluation of sample preparation methods, complementing traditional analytical performance parameters. While conventional extraction techniques (SPE, LLE, QuEChERS) still rely on solvents and reagents, generating large volumes of waste, emerging miniaturized approaches and methods employing green solvents, including NADES and SUPRAS, offer improved sustainability through reduced solvent consumption and safer chemical profiles. However, the adoption of green metrics in LC–MS food analysis remains limited, demonstrating the need for broader integration of sustainability assessment in this field.

## 7. Conclusions and Perspectives

The determination of exogenous toxic organic compounds in food remains analytically challenging due to their extreme chemical diversity, distinct physicochemical properties, and complex interactions with food matrices. These characteristics prevent the adoption of a universal analytical approach and instead require tailored extraction, cleanup, and chromatographic strategies adapted to specific contaminant classes. In this context, advanced LC–MS methodologies, particularly those incorporating HRMS, are essential to ensure reliable detection, identification, and quantification.

Marked differences among contaminant groups further highlight the need for chemically informed method development. PFASs are highly stable, persistent, and difficult to extract from lipid-rich matrices, whereas HAAs and PAHs and derivatives are mainly associated with thermal processing. Antibiotics and their metabolites exhibit wide polarity ranges and complex metabolic behavior, often requiring flexible analytical conditions. Across all contaminant classes, matrix–analyte interactions critically influence recovery, sensitivity, and selectivity, underscoring that a thorough understanding of the food matrix is as important as analyte characterization.

On the other hand, regulatory frameworks are continuously evolving toward stricter limits and expanded contaminant coverage, reinforcing the need for robust, sensitive, and adaptable analytical methods. At the same time, the relevance of bioaccumulation and contaminant transfer along the food chain highlights the importance of an integrated food safety perspective that links environmental contamination, food production, processing, and consumption.

The application of green analytical metrics provides a valuable framework for assessing the environmental sustainability of LC–MS-based food analysis. Metrics based on GAC and GSP principles allow objective comparison of sample preparation procedures beyond analytical performance alone. The assessment presented in this review indicates that conventional techniques such as solid-phase extraction, liquid–liquid extraction, and QuEChERS remain analytically effective, but are associated with significant environmental issues related to solvent consumption, waste generation, and multistep workflows. In contrast, microextraction approaches employing green solvents, including NADES and SUPRAS, demonstrate improved greenness through reduced solvent volumes and safer chemical profiles. Despite these advances, the use of green metrics in LC–MS-based food analysis remains limited, and dedicated tools for evaluating the synthesis and use of green solvents and advanced materials are still unavailable. Future efforts should focus on sustainable, multiresidue LC–MS and HRMS methodologies that integrate targeted and non-targeted strategies, supported by harmonized green metrics, to address current and emerging challenges in food safety.

## Figures and Tables

**Table 1:** Representative sample preparation methods used for detecting exogenous toxic organic compounds in food samples by LC-MS/MS.

Extraction Method	Sorbent/Solvent	Analyte	Matrix	LOD (μg kg^−1^)	LOQ (μg kg^−1^)	Recovery (%)	RSD (%)	Ref.
MSPE	COF	Antibiotics	Honey	1.0–75	4.0–228	70–115	<10	[118]
	COF	Antibiotics	Meat	0.1–1.0	0.3–3	82–110	<10	[116]
	COF	Antibiotics	Eggs	0.01–0.73	0.2–2.44	75–102	<10	[117]
	COF	Antibiotics	Eggs	0.009–0.272	0.029–0.908	70–120	<10	[119]
	COF	Antibiotics	Milk	0.004–0.044	0.013–0.136	61–103	<10	[120]
	Chitosan	Antibiotics	Milk	0.04–0.19	0.13–0.64	86–107	<10	[126]
	(HCP/Fe_3_O_4_)	Veterinary drugs	Milk	0.015–0.3	0.05–1	72–120	<20	[124]
	(HCP/Fe_3_O_4_)	Veterinary drugs	Honey	0.1–0.3	0.3–1.0	>85	<15	[129]
	Fe_3_O_4_@COF@Cys	HAAs	Meat	0.012–0.210	0.043–0.650	90–103	<10	[93]
	Fe_3_O_4_@PDA	HAAs	Meat	0.013–0.247	0.056–0.803	71–108	<10	[128]
	PEG-MWCNTs-MNP	Mycotoxins	Milk	0.005–0.050	0.015–0.150	82–106	<10	[125]
DSPE	Cation exchange	Veterinary drugs	Eggs	0.01–1.0	0.1–1.0	70–119	<20	[130]
	MWCNT	Antibiotics	Eggs	0.1–0.6	2.0	83–111	<20	[131]
	MIL-101(Cr)	Mycotoxins	Vegetable oil	1.5	5.0	94–102	<5	[132]
	MWCNT	Pesticides	Food	0.01–0.07	0.04–0.22	78–101	<10	[133]
MISPE	MIP	Veterinary drugs	Milk	0.34–2.21	1.12–7.36	95–98	<5	[134]
	MIP	Antibiotics	Milk	1.0–20	3.0–60	81–118	<15	[114]
PT-µSPE	Biochar	Pesticides	Fruits and vegetables	0.03–10	0.1–10	80–100	<20	[135]
	TAPT-BPDA	Antibiotics	Meat, eggs, milk	0.10–0.28	0.33–0.93	76–114	<5	[136]
ONLINE SPE	TPB-BFBIm-iCOF	PFASs	Seafood	<0.0017	0.0017	85–109	<10	[137]
QuEChERS	PSA, C18, ACN	Pesticides	Honey	0.1–3.0	5.0–10.0	70–120	<20	[138]
	PSA, C18, ACN	Pesticides	Tea, orange	0.07–0.29	0.7–10.0	90–109	<5	[139]
	PSA, C18, ACN	Pesticides	Meat	0.4–3.0	1.0–10.0	73–105	<10	[140]
	PSA, C18, ACN	Pesticides	Virgin olive oil	0.12	0.4	70–120	<20	[141]
	PSA, C18, ACN	Mycotoxins	Plant-based beverages	0.002–3	0.007–10	80–120	<20	[142]
	PSA, C18, ACN	Mycotoxins	Coffee beans	0.5–2	0.45–1	>65	<15	[143]
	C18, ACN, H_2_O	Mycotoxins	Grain products	0.001–20	0.015–60	61–109	<10	[144]
	PSA, C18, Z-Sep+, ACN	Pesticides, mycotoxins	Baby food	0.4–2	1.0–2.0	60–120	<20	[145]
QuEChERSER	ACN, H_2_O	PFASs	Beef, catfish, eggs	0.003–0.3	0.01–0.7	70–120	<20	[146]
QuEChUP	ACN, MeOH, EMR-Lipid	Pesticides and Veterinary drugs	Seafood	<0.001	<0.07	70–110	<20	[147]
sin-QuEChERS	MS/ddMS, ACN, FA	Pesticides	Green tea	1.0–2.0	2.0–10	74–111	<15	[148]
modified-QuEChERS	Chitosan, ACN	Pesticides and Veterinary drugs	Aquatic products	0.5–1.0	1.0–2.0	64–116	<20	[149]
DLLME	ACN, MeOH, ethyl acetate	Mycotoxins	Green tea	0.1–14	1.02–47-7	81–125	<15	[150]
	DES	Pesticides	Wine	0.00070–1.6	0.0024–5.0	56–100	<15	[151]
	NADES	Pesticides, PAHs	Eggs and meat	0.03–0.4	0.1–1.32	8–112	<20	[152]
DLLME-SFO	1-dodecanol, MeOH, ACN	Mycotoxins	Amaranthus seeds	0.07–0.73	0.22–0.9	80–100	<10	[153]
	NADES	Mycotoxins	Apple products	0.03–0.2	0.1–0.2	72–100	<15	[154]
VA-LLME	DES	Pesticides	Apples, pears, peaches, and grapes	0.08–0.3	2.1–2.9	97–100	<5	[155]
LLME	NADES	Mycotoxins	Vegetable oils	0.07–300	0.2–300	95–103	<10	[84]
SUPRAS	1-hexanol, H_2_O, THF	Mycotoxins	Orange juice, white bread, raisins, yogurt	0.01–2.1	0.41–2.97	60–107	<15	[156]

**Table 2:** Summary of existing metrics for analytical methods and applications. (software development and latest version update is provided).

Year	Name	Acronyms	Developers	Software	Reference
2002	National Environmental Methods Index	NEMI	U.S. Environmental Protection Agency (EPA).	NA *	[184]
2009	Green Assessment Profile	GAP	Raynie, D. and J. Driver.	NA	[185]
2011	Advanced national environmental methods index	Advanced NEMI	U.S. Environmental Protection Agency (EPA).	NA	[186]
2011	Analytical Method Volume Intensity	AMVI	Hartman, R., Helmy, R., Al-Sayah, M., & Welch, C. J.	NA	[187]
2011	High-Performance Liquid Chromatography Environmental Assessment Tool	HPLC-EAT	Gaber, Y., Törnvall, U., Kumar, M. A., Amine, M. A., & Hatti-Kaul, R.	HPLC-EAT program and user manual (Java program, v. 2011)	[188]
2012	Analytical Eco-Scale	Analytical Eco-Scale	Gałuszka, A., Konieczka, P., Migaszewski, Z. M., Namieśnik, J.	NA	[189]
2015	Green Certificate	Green Certificate	De la Guardia, M. & Armenta, S.,	NA	[190]
2016	Spider diagram	Spider diagram	Yang Shen, Chi Lo, D.R. Nagaraj, Raymond Farinato, Amy Essenfeld, P. Somasundaran	NA	[191]
2018	Green Analytical Procedure Index	GAPI	Płotka-Wasylka, J.	NA	[192]
2019	HEXAGON	HEXAGON	Ballester-Caudet, A., Campíns-Falcó, P., Pérez, B., Sancho, R., Lorente, M., Sastre, G., & González, C.	NA	[193]
2019	Analytical Method Greenness Score Calculator	AMGS	Hicks, M. B., Farrell, W., Aurigemma, C., Lehmann, L., Weisel, L., Nadeau, K., Lee, H., Moraff, C., Wong, M., Huang, Y., & Ferguson, P.	NA	[194]
2019	Red, Green and Blue Model	RGB	Nowak, M.P. & Kościelniak, P.	Template	[195]
2020	Analytical Greenness Calculator	AGREE	Pena-Pereira, F., Wojnowski, W., & Tobiszewski, M.	https://mostwiedzy.pl/AGREE (accessed on 22 December 2025; developed in Python 3.7, v. 2020)	[8]
2021	Complementary GAPI Tool	ComplexGAPI	Płotka-Wasylka, J. and Wojnowski, W.	https://mostwiedzy.pl/en/justyna-plotka-wasylka,647762-1/complexgapi (accessed on 22 December 2025, developed in Python 3 software, v. 2021)	[196]
2021	White Analytical Chemistry (WAC) Metrics	WAC-RGB	Nowak, P. M., Wietecha-Posłuszny, R., & Pawliszyn, J.	Template (Excel worksheets, 2021 last version)	[183]
2022	Analytical Greenness Metric for Sample Preparation	AGREEprep	Wojnowski, W., Tobiszewski, M., Pena-Pereira, F., & Psillakis, E.	https://mostwiedzy.pl/en/publication/agreeprep-analytical-greenness-metric-for-sample-preparation,157564-1 (accessed on 22 December 2025; user-friendly graphical interface (GUI), v. 2022)	[10]
2023	Sample preparation metric of sustainability	SPMS	González-Martín, R., Gutiérrez-Serpa, A., Pino, V., & Sajid, M.	Appendix (Not software-based, scoring done manually or in Excel)	[197]
2023	Blue Applicability Grade Index	BAGI	Manousi. N., Wojnowski, W., Płotka-Wasylkac, J., and Samanidou, V.	https://mostwiedzy.pl/en/publication/blue-applicability-grade-index-bagi-and-software-a-new-tool-for-the-evaluation-of-method-practicalit,161475-1 (accessed on 22 December 2025; metric applied via tables or spreadsheets)	[198]
2023	ChlorTox Scale	ChlorTox Scale	Nowak, P. M., Wietecha-Posłuszny, R., Płotka-Wasylka, J., & Tobiszewski, M	NA (Not software-based, scoring done manually or in Excel)	[199]
2024	Analytical Greenness Assessment Tool for Molecular Imprinting	AGREEmip	Marć, M., Wojnowski, W., Pena-Pereira, F. J., Tobiszewski, M., & Martín-Esteban, A.	https://mostwiedzy.pl/en/wojciech-wojnowski,174235-1/agreemip (accessed on 22 December 2025; developed in Python 3.9, v. 2024)	[200]
2024	Modified GAPI	MoGAPI	Mansour, F. R., Płotka-Wasylka, J. anD Locatelli, M.	https://bit.ly/MoGAPI (accessed on 22 December 2025; Web-based interactive tool, v. 2024)	[201]
2024	Complex Modified GAPI	ComplexMoGAPI	Mansour, F.R., Omer K. M., Płotka-Wasylka, J.	https://fotouhmansour.github.io/ComplexMoGAPI/ (accessed on 22 December 2025; Web-based interactive tool, v. 2024)	[202]
2024	Red, Green and Blue Model Fast	RGBfast	Nowak, P. M., & Arduini, F.	Excel template (v. 2024)	[203]
2025	RAPI Analytical Performance Index and Software	RAPI	Nowak, P. M., Wojnowski, W., Manousi, N., Samanidou, V., & Płotka-Wasylka, J.	https://rapi-index.anvil.app/ (accessed on 22 December 2025; Python-based software tool, v. 2024)	[204]
2025	Analytical Green Star Area	AGSA	Mansour, F. R., Bedair A., Belal, F., Magdy, G.	https://fotouhmansour.github.io/AGSA/ (accessed on 22 December 2025; free, open-source software tool, v. 2025)	[205]
2025	Greenness Evaluation Metric for Analytical Methods	GEMAM	Xin, T., Yu, L., Zhang, W., Guo, Y., Wang, C. and Li, Z.	https://gitee.com/xtDLUT/Gemam/releases/tag/Gemam-v1 (accessed on 22 December 2025; software developed in Python 3.11, v. 2025)	[206]
2025	Click Analytical Chemistry Index	CAC	Mansour, F. R., Bedair A., Locatelli, M.	https://bit.ly/CACI2025 (accessed on 22 December 2025; open-source tool, v. 2025)	[207]
2025	Graphical Layout Tool for Analytical Chemistry Evaluation	GLANCE	Fuente-Ballesteros, A., Jano, A., Ares, A. M., Valverde S. and Bernal, J.	bit.ly/409cwDd (accessed on 22 December 2025; Canva editable template, v. 2025)	[208]
2025	Red, Green and Blue Model ex ante	RGB ex ante	Nowak, P. M., Zima, A., Gołąb, M., & Woźniakiewicz, M.	Excel spreadsheet available on: https://ars.els-cdn.com/content/image/1-s2.0-S2772577424000946-mmc1.xlsx (accessed on 22 December 2025; v. 2025)	[209]
2025	Violet Innovation Grade Index	VIGI	Fuente-Ballesteros, A., Martínez-Martínez, V., Ares, A.M., Valverde, S., Samanidou, V., and Bernal, J.	https://bit.ly/VIGItool (accessed on 22 December 2025; free, open-access desktop application, v. 2025)	[210]

* NA: Not Available.

## Data Availability

The original contributions presented in this study are included in the article/Supplementary Materials. Further inquiries can be directed to the corresponding authors.

## Supplementary Materials

The following supporting information can be downloaded at https://www.mdpi.com/article/10.3390/foods15030517/s1, Figure S1: Distribution of publications (%) reporting major classes of exogenous toxic compounds across different food matrices, with bubble size indicating relative study frequency; Table S1: Regulatory Maximum Levels established for major chemical contaminant families in food: mycotoxins, veterinary drugs, antibiotics, non-polar pesticides, per- and polyfluoroalkyl substances, heterocyclic aromatic amines, and polycyclic aromatic hydrocarbons; Table S2: Overview of food matrices, major exogenous toxic compounds, matrix-related analytical challenges, and commonly applied extraction strategies reported in the literature.
