# Supplementary material for: Eco-Friendly Sample Preparation Trends for Exogenous Toxic Organic Compounds in Food: A Sustainable Perspective for LC-MS Analysis
Source: Foods. 2026 Feb 2;15(3):517. doi: 10.3390/foods15030517 (PMC12897172; doi:10.3390/foods15030517)
Supplement: Supplementary file 1 [file foods-15-00517-s001.zip › foods-4094040-supplementary.pdf]

Supplementary Material

Review

Eco-Friendly Sample Preparation Trends for Exogenous Toxic Organic Compounds in Food: A Sustainable Perspective for LC-MS Analysis

Mariel Cina [1,2], Alejandro Mandelli [2,3], María Del Valle Ponce [2,4], María Guiñez [2,3,*] and Soledad Cerutti [2,3,*]

[1] Instituto de Ciencias de La Tierra y Ambientales de La Pampa (INCITAP, CONICET-UNLPam), Facultad de Ciencias Exactas y Naturales, Universidad Nacional de La Pampa, Santa Rosa CP 6300, Argentina;

[2] Consejo Nacional de Investigaciones Científicas y Técnicas (CONICET), Godoy Cruz 2290,
Buenos Aires CP 1425, Argentina;

[3] Instituto de Química de San Luis (INQUISAL, CONICET-UNSL), Facultad de Química, Bioquímica y
Farmacia, Universidad Nacional de San Luis, Laboratorio de Espectrometría de Masas, Bloque III,
Ejército de Los Andes 950, San Luis CP 5700, Argentina

[4] Instituto de Física Aplicada (INFAP, CONICET-UNSL), Universidad Nacional de San Luis, Ejército de Los Andes 950, San Luis CP 5700, Argentina

* Correspondence: maevangelinaguinez@gmail.com (M.G.); ecerutti@gmail.com (S.C.)

Table S1. Regulatory Maximum Levels established for major chemical contaminant families in food: mycotoxins, veterinary drugs, antibiotics, non-polar pesticides, per- and polyfluoroalkyl substances, heterocyclic aromatic amines, and polycyclic aromatic hydrocarbons.

	FOOD MATRIX	MAXIMUM LEVEL OR STATUS	JURISDICTION / REGULATORY FRAMEWORK	REF.
**MYCOTOXINS**				
Aflatoxin B1	Cereals & derived products (except maize) for direct human consumption	**2 µg kg⁻¹**		
	Unprocessed maize	**5 µg kg⁻¹**		
	Cereal-based foods & baby foods for infants/young children; foods for special medical purposes (infants)	**0.10 µg kg⁻¹**		
	Spices (Piper spp., Myristica fragrans, Zingiber officinale, Curcuma longa)	**5 µg kg⁻¹**		
Total Aflatoxins (B1+B2+G1+G2)	Cereals & derived products (except maize) for direct consumption	**4 µg kg⁻¹**		
	Unprocessed maize	**10 µg kg⁻¹**		
	Spices (pepper, nutmeg, ginger, turmeric)	**10 µg kg⁻¹**		
Ochratoxin A	Unprocessed cereals	**5 µg kg⁻¹**		
	Cereal products for final consumption	**3 µg kg⁻¹**	EU — Reg. (EU) 2023/915; Annex I	
	Baby foods for infants/young children	**0.50 µg kg⁻¹**		[1]
	Spices (nutmeg, ginger, turmeric)	**15 µg kg⁻¹**		
	Capsicum spp. spices	**20 µg kg⁻¹**		
Deoxynivalenol	Unprocessed cereals (except durum wheat, maize, oats)	**1000 µg kg⁻¹**		
	Unprocessed durum wheat & maize	**1500 µg kg⁻¹**		
	Unprocessed oats (with husk)	**1750 µg kg⁻¹**		
	Cereals for the final consumer; popcorn maize	**750 µg kg⁻¹**		
	Bakery wares, cereal snacks & breakfast cereals	**400 µg kg⁻¹**		
	Pasta (dry)	**600 µg kg⁻¹**		
	Flours/semolina/bran/germ (retail)	**600 µg kg⁻¹**		
	Baby food & processed cereal-based foods (infants)	**150 µg kg⁻¹**		
Zearalenone	Unprocessed cereals (except maize)	**100 µg kg⁻¹**		

	Unprocessed maize	**350 µg kg^{-1}**		
	Cereal products for final consumption (flour, semolina, bran, germ)	**75 µg kg^{-1}**		
	Baby foods (infants/young children)	**20 µg kg^{-1}**		
Fumonisins (FB1+FB2)	Unprocessed maize	**4000 µg kg^{-1}**		
	Maize flour not for retail	**2000 µg kg^{-1}**		
	Other non-retail maize milling products	**1400 µg kg^{-1}**		
	Maize-based breakfast cereals & snacks	**800 µg kg^{-1}**		
	Maize products for final consumption	**1000 µg kg^{-1}**		
	Baby food & processed maize-based foods (infants)	**200 µg kg^{-1}**		
Trichothecenes (T-2 + HT-2)	Unprocessed cereal grains (general rule, except specific entries below)	**50 µg kg^{-1}**	EU — Reg. (EU) 2023/915 and 2024/1038; Annex I	[1,2]
	Unprocessed malting barley	**200 µg kg^{-1}**		
	Unprocessed barley (non-malting)	**150 µg kg^{-1}**		
	Unprocessed maize & durum wheat	**100 µg kg^{-1}**		
	Unprocessed oats (with husk)	**1250 µg kg^{-1}**		
	Cereals for the final consumer (excl. specific sub-entries)	**20 µg kg^{-1}**		
Aflatoxin M1	Raw/heat-treated milk & milk intended for dairy processing	**0.050 µg kg^{-1}**	EU — Reg. (EU) 2023/915; Annex I	[1]
	Infant formulae, follow-on formulae, foods for special medical purposes (infants)	**0.025 µg kg^{-1}**		
Patulin	Apple juice/nectar/purée	**50 µg kg^{-1}**	Codex — CXS 193-199	[3]
	Apple juice	**50 µg kg^{-1}**	FDA — CPG Sec. 510.150	[4]
Aflatoxins (sum)	Maize & maize products	**20 µg kg^{-1}**	FDA — CPG 555.400	[5]
Citrinin	Food supplements based on red yeast rice	**100 µg kg^{-1}**	EU — Reg. (EU) 2019/1901 and 2023/915; Annex I	[1,6]
**VETERINARY DRUGS**				
Nitrofurans (AOZ, AMOZ, AHD, SEM)	Meat, Milk, Eggs, Honey (all animal-origin foods)	MRPL **1 µg kg^{-1}** (enforcement threshold); Substance class prohibited	EU — Commission Decision 2003/181/EC (MRPLs); Reg. (EU) No 37/2010	[7, 8]
Chloramphenicol	Meat, Milk, Eggs, Honey	Prohibited	EU — Reg. (EU) No 37/2010	[8]
Dapsone				
Metronidazole	All food-producing animal products			

Florfenicol	Meat (swine)	**100 µg kg⁻¹** (MRL)	EU — Reg. (EU) No 37/2010, Annex I; Codex — Florfenicol MRLs (species-specific), FAO/WHO Veterinary Drug Index	[8, 9]
	Milk, Eggs	Prohibited	EU — Reg. (EU) No 37/2010	[8]
Erythromycin A	Meat (swine)	**200 µg kg⁻¹** (MRL)	EU — Reg. (EU) No 37/2010, Annex I; Codex — Erythromycin MRLs (species-specific), FAO/WHO Veterinary Drug Index	[8, 10]
	Milk	**40 µg kg⁻¹** (MRL)		
	Eggs	**150 µg kg⁻¹** (MRL)		
Tilmicosin	Meat (swine)	**50 µg kg⁻¹** (MRL)	EU — Reg. (EU) No 37/2010, Annex I; EMA/CVMP — Tilmicosin MRLs; Milk Extension Assessment Report	[8, 11]
	Milk	**50 µg kg⁻¹** (MRL)		
Tylosin A	Meat (swine)	**100 µg kg⁻¹** (MRL)	EU — Reg. (EU) No 37/2010; Codex — Tylosin MRLs (species-specific), FAO/WHO Veterinary Drug Index	[8, 12]
	Milk	**50 µg kg⁻¹** (MRL)		
	Eggs	**200 µg kg⁻¹** (MRL)		
Levamisole	Meat (swine)	**10 µg kg⁻¹** (MRL)	EU — Reg. (EU) No 37/2010, Annex I; Codex MRLs multiespecie; FDA tolerance: 21 CFR § 556.350)	[8, 13, 14]
**ANTIBIOTICS**				
Chloramphenicol	Foods of animal origin (muscle, liver, kidney, milk, eggs, honey; aquaculture)	**0.15 µg kg⁻¹** (RPA)	EU — Reg. (EU) 2019/1871 and 2021/808.	[15, 16]
Nitrofurans	Foods of animal origin (aquaculture, honey)	**0.5 µg kg⁻¹** (RPA)	EU — Reg. (EU) 2019/1871 and 2021/808	
Tetracyclines	Milk	**100 µg kg⁻¹** (MRL)	EU — Reg. (EU) 37/2010, Annex I; Codex CXL	[17, 18]
	Meat (muscle)	**100 µg kg⁻¹** (MRL)	EU — Reg. (EU) 37/2010, Annex I	[17]
	Liver	**300 µg kg⁻¹** (MRL)		
	Kidney	**600 µg kg⁻¹** (MRL)		
	Eggs	**200 µg kg⁻¹** (MRL)		
Sulfonamides	Meat (muscle, all food-producing species)	**100 µg kg⁻¹** (MRL)		

Benzylpenicillin (Penicillin G)	Milk	**4 µg kg⁻¹** (MRL)	EU — Reg. (EU) 37/2010, Annex I; FDA 21 CFR Part 556	[17, 19]
Amoxicillin		**4 µg kg⁻¹** (MRL)	EU — Reg. (EU) 37/2010, Annex I	[17]
Ampicillin		**4 µg kg⁻¹** (MRL)		
Ceftiofur	Meat (muscle / fat / liver / kidney)	**100 / 200 / 2000 / 6000 µg kg⁻¹** (MRLs)		
	Milk	**100 µg kg⁻¹** (MRL)		
Florfenicol	Liver	**3000 µg kg⁻¹** (MRL)		
Tylosin	Milk	**50 µg kg⁻¹** (MRL)		
Streptomycin + Dihydrostreptomycin	Kidney	**1000 µg kg⁻¹** (MRL)		
**NON-POLAR PESTICIDE**				
DDT (sum of isomers)	Apples / Citrus / Grapes / Tomato / Leafy / Oils / Cereals / Animal products	**0.01 mg kg⁻¹**	EU — Reg. (EC) 396/2005; Annex I (product list). Not listed in Annexes II–III → default; DG SANTE EU Pesticides Database	[20, 21]
Aldrin + Dieldrin (as dieldrin)	All listed matrices	**0.01 mg kg⁻¹**		
Endosulfan (sum of isomers + sulfate)		**0.01 mg kg⁻¹**		
Cypermethrins (sum of isomers)	Apple / Citrus / Grapes	**0.3 mg kg⁻¹** (MRL)	EU — Reg. (EC) 396/2005; Annex II-III	[20]
	Tomato / Leafy vegetables	**0.5 mg kg⁻¹** (MRL)		
	Oils / Cereals (wheat, maize, rice)	**0.2 mg kg⁻¹** (MRL)		
	Milk	**0.05 mg kg⁻¹** (CXL)	Codex CXS 193-1995; ML 0106 "Milks"	[22]
Deltamethrin (fat-soluble)	Apple / Citrus / Grapes	**0.2 mg kg⁻¹** (MRL)	E EU — Reg. (EC) 396/2005; Annex I (product list). Not listed in Annexes II–III → default	[21]
	Tomato / Leafy vegetables	**0.3 mg kg⁻¹** (MRL)		
	Maize (corn) grain	**0.7 mg kg⁻¹** (MRL)	EU — Reg. (EC) 396/2005; Annex II ; EFSA MRL Modification (2022)	[20, 23]
	Milk / Fat / Meat	**0.02 mg kg⁻¹** (MRL)	EU — Reg. (EC) 396/2005; Annex II-III	[21]
Permethrin (sum of isomers)	Apple / Citrus / Grapes	**0.2 mg kg⁻¹** (MRL)		
	Tomato / Leafy vegetables	**0.5 mg kg⁻¹** (MRL)		
	Milk	**0.05 mg kg⁻¹** (CXL)	Codex CXS 193-1995; ML 0106 "Milks"	[22]
Bifenthrin	Apple / Citrus / Grapes	**0.5 mg kg⁻¹** (MRL)	EU — Reg. (EC) 396/2005; Annex II-III; EU Pesticides Databas	[20, 21]
	Tomato / Leafy vegetables	**0.7 mg kg⁻¹** (MRL)		
	Milk / Meat / Fat	**0.02 mg kg⁻¹**		
λ-Cyhalothrin	Apple / Citrus / Grapes	**0.3 mg kg⁻¹** (MRL)		[20, 21, 24]
	Tomato / Leafy vegetables	**0.5 mg kg⁻¹** (MRL)		

Fenvalerate / Esfenvalerate	Fruits / Vegetables	**0.5 mg kg⁻¹** (MRL)	EU — Reg. (EC) 396/2005; Annex II-III; Annex II–III; EFSA Targeted MRL Review (2024)	
**PFAS**				
PFAS (PFOS + PFOA + PFNA + PFHxS)	Vegetables	No ML set; monitoring per recommendation	EU — Commission Recommendation (EU) 2022/1431	[25]
	Fruits			
	Oils			
	Cereals			
	Plant-based milks			
	Coffee / Tea			
PFOS	Meat (bovine/porcine/poultry)	**0.30 µg kg⁻¹** (ML)	EU — Reg. (EU) 2022/2388 and 2023/915	[26, 27]
PFOA		**0.80 µg kg⁻¹** (ML)	EU — Reg. (EU) 2022/2388	[26]
PFNA		**0.20 µg kg⁻¹** (ML)		
PFHxS		**0.20 µg kg⁻¹** (ML)		
PFAS (PFOS + PFOA + PFNA + PFHxS)		**1.3 µg kg⁻¹** (ML)		
PFOS / PFOA / PFNA / PFHxS	Milk and Dairy Products (incl. cheese / yogurt)	No ML set; monitoring per recommendation	EU — Commission Recommendation (EU) 2022/1431	[25]
PFOS	Eggs	**1.0 µg kg⁻¹** (ML)	EU — Reg. (EU) 2022/2388	[26]
PFOA		**0.30 µg kg⁻¹** (ML)		
PFNA		**0.70 µg kg⁻¹** (ML)		
PFHxS		**0.30 µg kg⁻¹** (ML)		
PFAS (PFOS + PFOA + PFNA + PFHxS)		**1.7 µg kg⁻¹ (ML)**		
PFOS / PFOA / PFNA / PFHxS	Processed Foods (plant / animal)	No ML set; monitoring per recommendation	EU — Commission Recommendation (EU) 2022/1431	[25]
PFOS	Fish (general species)	**2.0 µg kg⁻¹** (ML)	EU — Reg. (EU) 2022/2388	[26]
PFOA		**0.20 µg kg⁻¹** (ML)		
PFNA		**0.50 µg kg⁻¹** (ML)		
PFHxS		**0.20 µg kg⁻¹** (ML)		
PFOS	Fish (listed species group 1)	**7.0 µg kg⁻¹** (ML)		
PFOS	Fish (listed species group 2)	**35 µg kg⁻¹** (ML)		
PFOS	Crustaceans & bivalve molluscs	**3.0 µg kg⁻¹** (ML)		
PFOA		**0.70 µg kg⁻¹** (ML)		
PFHxS		**1.5 µg kg⁻¹** (ML)		
PAFS (PFOS + PFOA + PFNA + PFHxS)		**5.0 µg kg⁻¹** (ML)		
PFAS (PFOS + PFOA + PFNA + PFHxS)	General dietary exposure benchmark	**4.4 ng kg⁻¹** (TWI)	EFSA Scientific Opinion (2020)	[27]

**HAAs**				
IQ	Cooked meats (grilled/roasted/pan-fried; beef, pork, poultry)	No ML in food; classified as Group 2A (probably carcinogenic to humans)	IARC Monographs; Not listed with MLs in EU Reg. (EU) 2023/915	[28]
MeIQx	Cooked meats	No ML; Group 2B (possibly carcinogenic); listed under California Prop 65 (carcinogen)	IARC Monographs; OEHHA Prop 65 listing; Not listed with MLs in EU Reg. (EU) 2023/915	[29]
PhIP	Cooked meats (and processed meats)	No ML; Group 2B (possibly carcinogenic); Prop 65 carcinogen	IARC Monographs; OEHHA Prop 65 listing/database; Not listed with MLs in EU Reg. (EU) 2023/915	[30]
AαC / MeAαC	High-temperature cooked meats (≥300 °C; grilling/charcoal)	No ML; several Group 2B (possibly carcinogenic)	IARC Monographs; Not listed with MLs in EU Reg. (EU) 2023/915	[31]
7,8-DiMeIQx / 4,8-DiMeIQx / IQx / MeIQ	Cooked meats	No ML; IARC evaluations place multiple HAAs in Group 2B		
**PAHs**				
BaP	Edible oils and fats	**2 μg kg⁻¹** (ML)	EU — Regulation (EC) No 1881/2006 and 835/2011	[32]
PAH4 (BaP + BaA + BbF + CHR)		**10 μg kg⁻¹** (ML)		
BaP	Coconut oil	**2 μg kg⁻¹** (ML)		
Total PAHs		**20 μg kg⁻¹** (ML)		
BaP	Oils (general category)	**10 μg kg⁻¹** (ML)		
	Smoked meat and meat products	**2 μg kg⁻¹** (ML)		
PAH4 (BaP + BaA + BbF + CHR)		**12 μg kg⁻¹** (ML)		
BaP	Cocoa products	**5 μg kg⁻¹** (ML)		
PAH4 (BaP + BaA + BbF + CHR)		**30 μg kg⁻¹** (ML)		
BaP	Smoked seafood	**5 μg kg⁻¹** (ML)		
PAH4 (BaP + BaA + BbF + CHR)		**30 μg kg⁻¹** (ML)		
BaP	Bivalve mollusks	**5 μg kg⁻¹** (ML)		
PAH4 (BaP + BaA + BbF + CHR)		**30 μg kg⁻¹** (ML)		
BaP	Infant foods (cereals, formulas, dietetic)	**1 μg kg⁻¹** (ML)		
BaP	Mollusks and crustaceans	**18.0 μg kg⁻¹** (ML)	Brazil — ANVISA (2019)	[33]

PAH4 (BaP + BaA + BbF + CHR)	Infant foods (cereals, formulas, dietetic)	**1 µg kg⁻¹** (ML)	EU — Regulation (EC) No 1881/2006	[32]
BaP	Meat	**5 µg kg⁻¹** (ML)	China — GB 2762–2012	[34]
	Fish	**6.0 µg kg⁻¹** (ML)	Brazil — ANVISA (2019) and EU — Regulation (EU) No 1146/2014	[33, 35]
9,10-anthraquinone	Meat	**10 µg kg⁻¹** (ML)	EU — Regulation (EU) No 1146/2014	[35]
BaP	General foods	ML established (indicator compound; marker of carcinogenic exposure)	EU — Regulation (EU) No 835/2011; EFSA Scientific Opinion (2020)	[36]
PAH4 (BaP + BaA + BbF + CHR)		ML established (indicator of PAH contamination)	EU — Regulation (EU) No 835/2011; EFSA Scientific Opinion (2020)	[32, 36]

ML (*Maximum Level*): Maximum permitted concentration of a contaminant (e.g., mycotoxins, PAHs, HAAs) in a food commodity, expressed in µg kg⁻¹ or mg kg⁻¹, established under Regulation (EC) No 1881/2006 (now consolidated as Regulation (EU) 2023/915) and corresponding Codex or national standards.

MRL (*Maximum Residue Limit*): Maximum legally permitted concentration of a residue of a pesticide or veterinary drug in food of plant or animal origin, derived from authorized agricultural or veterinary use, defined in Regulation (EC) No 396/2005 (pesticides) and Regulation (EU) No 37/2010 (veterinary medicinal products).

MRPL (*Minimum Required Performance Limit*): Minimum level of performance that laboratories must achieve when confirming the presence of a prohibited or non-authorized substance (e.g., chloramphenicol, nitrofurans), used for official control according to Commission Decision 2002/657/EC and Regulation (EU) No 2019/1871.

RPA (*Reference Point for Action*): Concentration threshold used by control authorities to trigger enforcement action for non-authorized pharmacologically active substances, as defined in Regulation (EU) 2019/1871 and Implementing Regulation (EU) 2021/808.

TWI (*Tolerable Weekly Intake*): Estimated maximum amount of a contaminant or toxic compound that can be ingested weekly over a lifetime without appreciable health risk, expressed in ng·kg⁻¹ body weight per week. Applied notably by EFSA (2020) for PFAS (sum of PFOS, PFOA, PFNA, PFHxS).

CXL (*Codex Maximum Level*): Internationally recommended maximum level or residue limit established by the Codex Alimentarius Commission (CXS 193-1995, Rev. 2019), harmonized with FAO/WHO risk assessments.

PAH4 (*Sum of 4 Polycyclic Aromatic Hydrocarbons*): Sum of benzo[a]pyrene (BaP), benz[a]anthracene (BaA), benzo[b]fluoranthene (BbF), and chrysene (CHR) — used as indicator group for total PAH contamination according to Reg. (EU) No 835/2011.

Table S2. Overview of food matrices, major exogenous toxic compounds, matrix-related analytical challenges, and commonly applied extraction strategies reported in the literature.

Food Matrix	Major Exogenous Toxic Compounds	Key Components of the Matrix	Challenges in Analytical Chemistry	Principal Extraction Techniques	References
Fruits and vegetables	Pesticides, Mycotoxins, and Antibiotics	Sugars and pectins	Matrix effect	QuEChERS, LLE/SPE	[39,47-49,54,55,65]
Cereals and flours	Mycotoxins.	Sugars, starches and proteins	Matrix effect. Rigorous cleanup is required	QuEChERS and SPE	[41-46,52,64]
Oils	Pesticides, Mycotoxins, and PAH derivatives	Fatty acids	Low recovery (<50%) of non-polar compounds due to high lipid content	SPE, LLE. QuEChERS	[45,51]
Plant-based milks	Pesticides, Mycotoxins, HAAs, and PAH derivatives.	Sugars, fats and proteins	Matrix effect. Difficulty in separating phases. Requires purification and/or pre-concentration.	QuEChERS, LLE/SPE	[50,53]
Milk	Pesticides, Antibiotics, Veterinary drugs, Mycotoxins, and PFAS	Water, fats, proteins and sugars	Variability in physicochemical properties of analytes. Requires selective extraction	SPE	[38,48,56-62,66-70]
Meat	Pesticides, Mycotoxins, Antibiotics, Veterinary drugs, PFAS, HAAs, and PAH derivatives	Proteins and fats	Uniform recoveries in multi-residue methods. Requires rigorous deproteinization and phase separation procedures	SPE, QuEChERS	[40,48,51,53,56-62,69,70]
Eggs	Pesticides, Antibiotics, Veterinary drugs, and PFAS.	Water, proteins and fats	Complex development of multi-class extraction. Requires highly effective cleanup	SPE	[37,48,60,63,68,70]
Honey	Pesticides, Antibiotics, Veterinary drugs, and PAH derivatives.	Sugars and water	Significant ion suppression in MS. Difficulty in multi-class detection	SPE, QuEChERS	[48,56,66,67]

Dairy products	Pesticides, Antibiotics, Veterinary drugs, PAH derivatives, and PFAS	Fats and proteins	Co-extraction of impurities. Rigorous Deproteinization.	SPE, QuEChERS	[38,48,56-62,66-70]

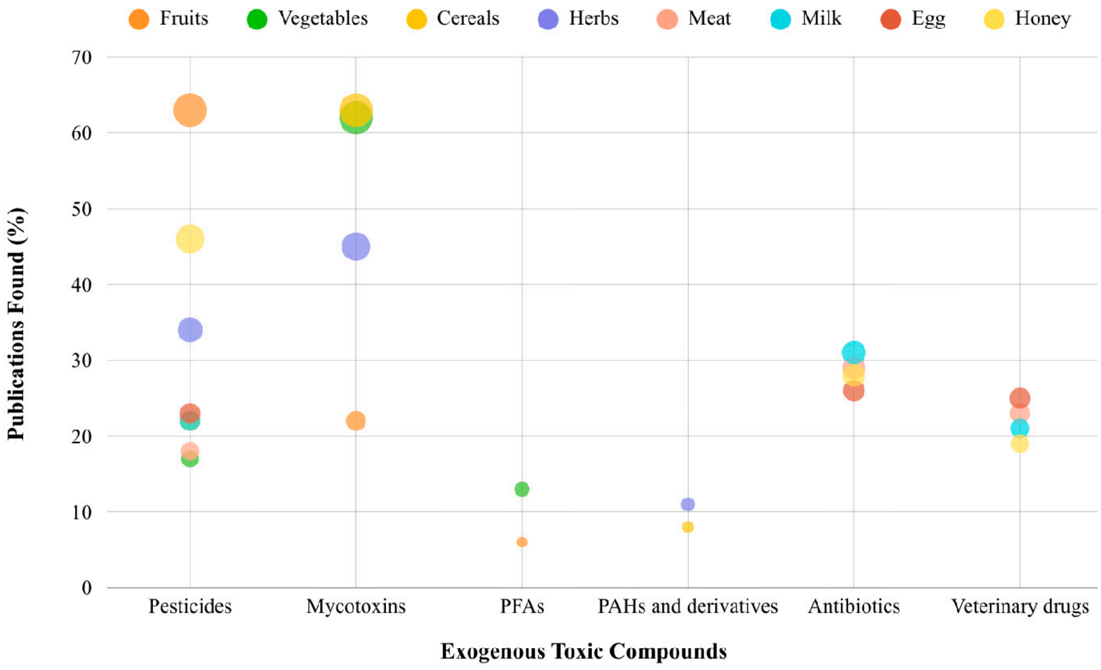

Figure S1. Distribution of publications (%) reporting major classes of exogenous toxic compounds across different food matrices, with bubble size indicating relative study frequency.

References Supplementary Material

[1] European Union. *Commission Regulation (EU) 2023/915 of 25 April 2023 on Maximum Levels for Certain Contaminants in Food and repealing Regulation (EC) No 1881/2006* (consolidated versions consulted). *Off. J. Eur. Union* **2023**, *L119*, 103–157. Available online: https://eur-lex.europa.eu/legal-content/EN/TXT/?uri=CELEX:32023R0915 (Accessed on 23 November 2025).

[2] European Union. *Commission Regulation (EU) 2024/1038 of 9 April 2024 amending Regulation (EU) 2023/915 as regards maximum levels of T-2 and HT-2 toxins in food. Off. J. Eur. Union* **2024**. Available online: https://eur-lex.europa.eu/legal-content/EN/TXT/?uri=CELEX:32024R1038 (Accessed on 23 November 2025).

[3] Codex Alimentarius Commission. *General Standard for Contaminants and Toxins in Food and Feed (CXS 193-1995, Rev. 2024).* FAO/WHO: Rome, Italy, 2024. Available online: https://www.fao.org/fao-who-codexalimentarius/codex-texts/list-standards/en/ (Accessed on 23 November 2025).

[4] U.S. Food and Drug Administration (FDA). *Compliance Policy Guide Sec. 510.150—Apple Juice, Apple Juice Concentrates and Apple Juice Products; Adulteration—Patulin.* FDA: Silver Spring, MD, USA, 2018. Available online: https://www.fda.gov/media/121201/download (Accessed on 23 November 2025).

[5] U.S. Food and Drug Administration (FDA). *Compliance Policy Guide Sec. 555.400—Aflatoxins in Human Food.* FDA: Silver Spring, MD, USA, 2021. Available online: https://www.fda.gov/media/121202/download (Accessed on 23 November 2025).

[6] European Union. *Commission Regulation (EU) 2019/1901 of 7 November 2019 amending Regulation (EC) No 1881/2006 as regards maximum levels of citrinin in food supplements based on rice fermented with Monascus purpureus. Off. J. Eur.*

Union **2019**. Available online: https://eur-lex.europa.eu/legal-content/EN/TXT/?uri=CELEX:32019R1901 (Accessed on 23 November 2025).

[7] European Commission. *Commission Decision 2003/181/EC of 13 March 2003 amending Decision 2002/657/EC as regards the setting of minimum required performance limits (MRPLs) for certain residues in food of animal origin. Off. J. Eur. Union* **2003**, *L71*, 17–18. Available online: https://eur-lex.europa.eu/legal-content/EN/TXT/?uri=CELEX:32003D0181 (Accessed on 23 November 2025).

[8] European Union. *Commission Regulation (EU) No 37/2010 of 22 December 2009 on pharmacologically active substances and their classification regarding maximum residue limits in foodstuffs of animal origin* (latest consolidated version consulted). *Off. J. Eur. Union* **2010**, *L15*, 1–72. Available online: https://eur-lex.europa.eu/legal-content/EN/TXT/?uri=CELEX:32010R0037 (Accessed on 23 November 2025).

[9] Codex Alimentarius Commission. *Veterinary Drug Detail—Florfenicol (MRLs by species and tissue).* FAO/WHO: Rome, Italy. Available online: https://www.fao.org/fao-who-codexalimentarius/codex-texts/dbs/vetdrugs/en/ (Accessed on 23 November 2025).

[10] Codex Alimentarius Commission. *Veterinary Drug Detail—Erythromycin (MRLs by species and tissue).* FAO/WHO: Rome, Italy. Available online: https://www.fao.org/fao-who-codexalimentarius/codex-texts/dbs/vetdrugs/en/ (Accessed on 23 November 2025).

[11] European Medicines Agency (EMA), Committee for Medicinal Products for Veterinary Use (CVMP). *Tilmicosin—Milk Extension: Summary Report.* EMA: London, UK. Available online: https://www.ema.europa.eu/en/documents (Accessed on 23 November 2025).

[12] Codex Alimentarius Commission. *Veterinary Drug Detail—Tylosin (MRLs by species and tissue).* FAO/WHO: Rome, Italy. Available online: https://www.fao.org/fao-who-codexalimentarius/codex-texts/dbs/vetdrugs/en/ (Accessed on 23 November 2025).

[13] Codex Alimentarius Commission. *Veterinary Drug Detail—Levamisole (MRLs by species and tissue).* FAO/WHO: Rome, Italy. Available online: https://www.fao.org/fao-who-codexalimentarius/codex-texts/dbs/vetdrugs/en/ (Accessed on 23 November 2025).

[14] U.S. Food and Drug Administration (FDA). *21 CFR § 556.350—Levamisole: Tolerances for residues of new animal drugs in foods.* FDA: Silver Spring, MD, USA. Available online: https://www.ecfr.gov/current/title-21/part-556 (Accessed on 23 November 2025).

[15] European Union. *Commission Regulation (EU) 2019/1871 of 7 November 2019 concerning reference points for action for non-authorized pharmacologically active substances present in food of animal origin and repealing Decision 2005/34/EC; consolidated versions consulted. Off. J. Eur. Union* **2019**, *L289*, 41–49. Available online: https://eur-lex.europa.eu (Accessed on 23 November 2025).

[16] European Union. *Commission Implementing Regulation (EU) 2021/808 on the performance of analytical methods and on the interpretation of results for residues of pharmacologically active substances in food-producing animals; and on the methods to be used for sampling. Off. J. Eur. Union* **2021**, *L180*, 84–109. Available online: https://eur-lex.europa.eu (Accessed on 23 November 2025).

[17] Codex Alimentarius Commission. *Maximum Residue Limits (MRLs) and Risk Management Recommendations for Veterinary Drugs (CXM 2-2024).* FAO/WHO:

Rome, Italy, 2024. Available online: https://www.fao.org/fao-who-codexalimentarius/codex-texts/list-standards/en/ (Accessed on 23 November 2025).

[18] U.S. Food and Drug Administration (FDA). *21 CFR Part 556—Tolerances for Residues of New Animal Drugs in Food.* U.S. FDA: Silver Spring, MD, USA. Available online: https://www.ecfr.gov/current/title-21/part-556 (Accessed on 23 November 2025).

[19] European Union. *Regulation (EC) No 396/2005 on maximum residue levels of pesticides in or on food and feed of plant and animal origin (consolidated). Off. J. Eur. Union* **2005**, *L70*, 1–16. Available online: https://food.ec.europa.eu/plants/pesticides/eu-pesticides-database_en (Accessed on 23 November 2025).

[20] European Commission (DG SANTE). *EU Pesticides—Default MRL rule (0.01 mg/kg) and Annex listings.* Available online: https://food.ec.europa.eu/plants/pesticides/eu-pesticides-database_en (Accessed on 23 November 2025).

[21] European Commission. *EU Pesticides Database—MRLs (Annex II–III); commodity-specific values.* Available online: https://food.ec.europa.eu/plants/pesticides/eu-pesticides-database_en (Accessed on 23 November 2025).

[22] Codex Alimentarius Commission (FAO/WHO). *CXS 193-1995—General Standard for Contaminants in Food and Feed; ML 0106 (Milks)—Cypermethrins & Permethrin.* Available online: https://www.fao.org/fao-who-codexalimentarius (Accessed on 23 November 2025).

[23] EFSA. *Modification of existing MRL for deltamethrin in maize/corn. EFSA J.* **2022**, *20*(7), e07446. Available online: https://www.efsa.europa.eu (Accessed on 23 November 2025).

[24] EFSA. *Targeted risk assessment of MRLs for λ-Cyhalothrin. EFSA J.* **2024**, *22*(6), e08816. Available online: https://www.efsa.europa.eu (Accessed on 23 November 2025).

[25] European Commission. *Commission Recommendation (EU) 2022/1431 of 24 August 2022 on the monitoring of perfluoroalkyl substances in food. Off. J. Eur. Union* **2022**, *L221*, 105–109. Available online: https://eur-lex.europa.eu (Accessed on 23 November 2025).

[26] European Union. *Commission Regulation (EU) 2022/2388 of 7 December 2022 amending Regulation (EC) No 1881/2006 as regards maximum levels of perfluoroalkyl substances in certain foodstuffs. Off. J. Eur. Union* **2022**, *L316*, 38–41. Available online: https://eur-lex.europa.eu (Accessed on 23 November 2025).

[27] EFSA CONTAM Panel. *Risk to Human Health Related to the Presence of Perfluoroalkyl Substances in Food. EFSA J.* **2020**, *18*(9), 6223. Available online: https://www.efsa.europa.eu/en/efsajournal/pub/6223 (Accessed on 23 November 2025).

[28] Bulanda, S.; Szumska, M.; Nowak, A.; Janoszka, B.; Damasiewicz-Bodzek, A. *Determination of Polar Heterocyclic Aromatic Amines in Food by UPLC–MS/MS: Occurrence and Toxicological Overview. Foods* **2025**, *14*, 559. https://doi.org/10.3390/foods14040559.

[29] National Toxicology Program (NTP). *Selected Heterocyclic Amines: PhIP, MeIQ, and MeIQx. Background on IARC Group 2A–2B evaluations.* 2002. Available online: https://ntp.niehs.nih.gov (Accessed on 23 November 2025).

[30] California Environmental Protection Agency (OEHHA). *MeIQx—Listed under Proposition 65 (Carcinogen)*. Available online: https://oehha.ca.gov/proposition-65 (Accessed on 23 November 2025).

[31] California Environmental Protection Agency (OEHHA). *Prop 65 Chemical Database (includes PhIP, MeIQx, etc.).* Available online: https://oehha.ca.gov/proposition-65 (Accessed on 23 November 2025).

[32] European Commission. *Commission Regulation (EU) No 835/2011 of 19 August 2011 amending Regulation (EC) No 1881/2006 as regards maximum levels for Polycyclic Aromatic Hydrocarbons in foodstuffs. Off. J. Eur. Union* **2011**, *L215*, 4–8. Available online: https://eur-lex.europa.eu/legal-content/EN/TXT/?uri=CELEX:32011R0835 (Accessed on 23 November 2025).

[33] Brazilian Health Regulatory Agency (ANVISA). *Resolution RDC No. 281/2019. Establishes provisional concern levels for BaP equivalents in fish, mollusks, and crustaceans.* Available online: https://www.gov.br/anvisa (Accessed on 23 November 2025).

[34] National Health Commission of the People's Republic of China. *Maximum Levels of Contaminants in Foods (GB 2762–2012).* Beijing, China, 2012. Available online: http://www.nhc.gov.cn (Accessed on 23 November 2025).

[35] European Union. *Regulation (EU) No 1146/2014 amending Annexes II and III to Regulation (EC) No 396/2005 as regards maximum residue levels for anthraquinone in meat and related matrices. Off. J. Eur. Union* **2014**, *L308*, 1–6. Available online: https://eur-lex.europa.eu/legal-content/EN/TXT/?uri=CELEX:32014R1146 (Accessed on 23 November 2025).

[36] European Food Safety Authority (EFSA). *Scientific Opinion on Polycyclic Aromatic Hydrocarbons (PAHs) in Food—Assessment of Occurrence and Toxicological Relevance. EFSA J.* **2020**, *18*(4), e06177. Available online: https://www.efsa.europa.eu/en/efsajournal/pub/6177 (Accessed on 23 November 2025).

[37] Paoletti, F.; Sdogati, S.; Barola, C.; Giusepponi, D.; Moretti, S.; Galarini, R. Development and validation of a multiclass confirmatory method for the determination of over 60 antibiotics in eggs using liquid-chromatography high-resolution mass spectrometry. *Food Control* **2021**, *127*, 108109. https://doi.org/10.1016/j.foodcont.2021.108109

[38] Kiszkiel-Taudul, I.; Stankiewicz, P. Microextraction of tigecycline using deep eutectic solvents and its determination in milk by LC-MS/MS method. *Journal of Agricultural and Food Chemistry* **2023**, *71*(30), 11716–11725. https://doi.org/10.1021/acs.jafc.3c03023

[39] Tong, W.; Huang, R.; Zuo, H.; Zarabadipour, C.; Moore, A.; Hamel, D.; Letendre, L. Feasibility of establishing a veterinary marker to total residue in edible tissues with non-radiolabeled study using high-resolution mass spectrometry. *Research in Veterinary Science* **2022**, *149*, 60–70. https://doi.org/10.1016/j.rvsc.2022.06.006

[40] Liu, X.; Wang, X.; Hong, S.; Zhou, H.; Cao, X.; Li, K.; Rao, Q. A novel approach based on supramolecular solvents microextraction for quick detection of perfluoroalkyl acids and their precursors in aquatic food. *Journal of Hazardous Materials* **2024**, *480*, 136169. https://doi.org/10.1016/j.jhazmat.2024.136169

[41] Gab-Allah, M.A.; Tahoun, I.F.; Yamani, R.N.; Rend, E.A.; Shehata, A.B. Eco-friendly and sensitive analytical method for determination of T-2 toxin and HT-2 toxin in cereal products using UPLC-MS/MS. *Journal of Food Composition and Analysis* **2022**, *107*, 104395. https://doi.org/10.1016/j.jfca.2022.104395

[42] Giannioti, Z.; Albero, B.; Hernando, M.D.; Bontempo, L.; Pérez, R.A. Determination of regulated and emerging mycotoxins in organic and conventional gluten-free flours by LC-MS/MS. *Toxins* **2023**, *15*(2), 155. https://doi.org/10.3390/toxins15020155

[43] Cina, M.; del Valle Ponce, M.; Fernandez, L.; Cerutti, S. A green approach for Ochratoxin A determination in coffee infusions. *Journal of Food Composition and Analysis* **2022**, *114*, 104777. https://doi.org/10.1016/j.jfca.2022.104777

[44] Ponce, M.D.V.; Cina, M.; López, C.; Cerutti, S. Polyurethane Foam as a Novel Material for Ochratoxin A Removal in Tea and Herbal Infusions—A Quantitative Approach. *Foods* **2023**, *12*(9), 1828. https://doi.org/10.3390/foods12091828

[45] Pradanas-González, F.; Aragoneses-Cazorla, R.; Merino-Sierra, M.Á.; Andrade-Bartolomé, E.; Navarro-Villoslada, F.; Benito-Pena, E.; Moreno-Bondi, M.C. Extracting mycotoxins from edible vegetable oils by using green, ecofriendly deep eutectic solvents. *Food Chemistry* **2023**, *429*, 136846. https://doi.org/10.1016/j.foodchem.2023.136846

[46] Pradanas-González, F.; Álvarez-Rivera, G.; Benito-Peña, E.; Navarro-Villoslada, F.; Cifuentes, A.; Herrero, M.; Moreno-Bondi, M.C. Mycotoxin extraction from edible insects with natural deep eutectic solvents: a green alternative to conventional methods. *Journal of Chromatography A* **2021**, *1648*, 462180. https://doi.org/10.1016/j.chroma.2021.462180

[47] Lawal, A.; Low, K. Residual determination of multiple pesticides in vegetable samples by LC-MS/MS coupled with modified QuEChERS-dSPE ionic liquid-based DLLME method. *Journal of the Turkish Chemical Society Section A: Chemistry* **2021**, *8*(2), 693–704. https://doi.org/10.18596/jotcsa.845578

[48] Regulation - 149/2008 - EN - EUR-LEx. Available online: https://eur-lex.europa.eu/eli/reg/2008/149/oj (accessed on November 2025).

[49] Sivakumar, S.; Angappan, S.; Thiyagarajan, E.; Sankaran, S.P.; Perumal, R.; Veeranan, V.A.G.; Ikram, M. Study of dissipation dynamics and persistent toxicity

of selected insecticides in chilli using LCMSMS. *Scientific Reports* **2025**, *15*(1), 3585. https://doi.org/10.1038/s41598-025-86724-2

[50] Mandelli, A.; Bochetto, A.; Guiñez, M.; Cerutti, S. Quantitative Analysis and Health Risk Assessment of Heterocyclic Aromatic Amines in Plant-Based Milk Beverages. *Foods* **2025**, *14*(19), 3295. https://doi.org/10.3390/foods14193295

[51] Sonego, E.; Bhattarai, B.; Duedahl-Olesen, L. Detection of nitrated, oxygenated and hydrogenated polycyclic aromatic compounds in smoked fish and meat products. *Foods* **2022**, *11*(16), 2446. https://doi.org/10.3390/foods11162446

[52] Vera-Baquero, F.L.; Pérez-Quintanilla, D.; Morante-Zarcero, S.; Sierra, I. Assessment of atropine and scopolamine in commercial multigrain cereal-based baby products using UHPLC-TQ-MS/MS and solid phase extraction with MCM-41 mesostructured silica as sorbent. *Food Chemistry* **2025**, *472*, 142875. https://doi.org/10.1016/j.foodchem.2025.142875

[53] Feng, Y.; Shi, Y.; Huang, R.; Wang, P.; Li, G. Simultaneous detection of heterocyclic aromatic amines and acrylamide in thermally processed foods by magnetic solid-phase extraction combined with HPLC-MS/MS based on cysteine-functionalized covalent organic frameworks. *Food Chemistry* **2023**, *424*, 136349. https://doi.org/10.1016/j.foodchem.2023.136349

[54] Merlo, F.; Centenaro, D.; Maraschi, F.; Profumo, A.; Speltini, A. Green and efficient determination of fluoroquinolone residues in edible green fruits and leafy vegetables by ultrasound-assisted extraction followed by HPLC-MS/MS. *Molecules* **2022**, *27*(19), 6595. https://doi.org/10.3390/molecules27196595

[55] Notardonato, I.; Gianfagna, S.; Castoria, R.; Ianiri, G.; De Curtis, F.; Russo, M.V.; Avino, P. Critical review of the analytical methods for determining the mycotoxin

patulin in food matrices. *Reviews in Analytical Chemistry* **2021**, *40*(1), 144–160. https://doi.org/10.1515/revac-2021-0131

[56] Mehl, A.; Hudel, L.; Bücker, M.; Morlock, G.E. Validated screening method for 81 multiclass veterinary drug residues in food via online-coupling high-throughput planar solid-phase extraction to high-performance liquid chromat[67]ography–orbitrap tandem mass spectrometry. *Journal of Agricultural and Food Chemistry* **2022**, *70*(35), 10886–10898. https://doi.org/10.1021/acs.jafc.2c03925

[57] Sun, Q.; Liu, J.; Gou, Y.; Chen, T.; Shen, X.; Wang, T.; Hua, Y. Determination of veterinary drugs in foods of animal origin by QuEChERS coupled with ultra performance liquid chromatography-tandem mass spectrometry (UPLC-MS/MS). *Journal of Chromatography A* **2025**, *1744*, 465726. https://doi.org/10.1016/j.chroma.2025.465726

[58] Khaled, O.; Ryad, L.; Nagi, M.; Eissa, F. Multiclass method for detecting 41 antibiotic residues in bovine liver, muscle, and milk using LC-Q-Orbitrap-HRMS. *Journal of Food Composition and Analysis* **2024**, *132*, 106299. https://doi.org/10.1016/j.jfca.2024.106299

[59] Li, Y.; Zhou, W.; Jiang, R.W.; Pawliszyn, J. Solid-phase microextraction with recessed matrix compatible coating for in situ sampling of per-and polyfluoroalkyl substances in meat. *Food Chemistry* **2025**, *480*, 143891. https://doi.org/10.1016/j.foodchem.2025.143891

[60] Lavrukhina, O.I.; Amelin, V.G.; Kish, L.K.; Tretyakov, A.V.; Pen'kov, T.D. Determination of residual amounts of antibiotics in environmental samples and food products. *Journal of Analytical Chemistry* **2022**, *77*(11), 1349–1385. https://doi.org/10.1134/S1061934822110077

[61] de Freitas, L.V.P.; Alponti, A.L.B.; Campanharo, S.C.; Damaceno, M.A.; da Silva, A.F.B.; de Oliveira Souza, M.C.; Paschoal, J.A.R. QuEChERS vs QuEChERS-DLLME: Toward an eco-friendly, selective, and sensitive method for levamisole determination in fish. *Microchemical Journal* **2025**, 114524. https://doi.org/10.1016/j.microc.2025.114524

[62] Al-Shaalan, N.H.; Nasr, J.J.; Shalan, S.; El-Mahdy, A.M. Use of green-modified micellar liquid chromatography for the determination of imidocarb dipropionate residues in food samples. *Microchemical Journal* **2022**, *178*, 107316. https://doi.org/10.1016/j.microc.2022.107316

[63] Teglia, C.M.; Guiñez, M.; Culzoni, M.J.; Cerutti, S. Determination of residual enrofloxacin in eggs due to long term administration to laying hens. Analysis of the consumer exposure assessment to egg derivatives. *Food Chemistry* **2021**, *351*, 129279. https://doi.org/10.1016/j.foodchem.2021.129279

[64] Jonard, C.; Chandelier, A.; Eylenbosch, D.; Pannecoucque, J.; Godin, B.; Douny, C.; Gofflot, S. Multi-Mycotoxin Analyses by UPLC-MS/MS in Wheat: The Situation in Belgium in 2023 and 2024. *Foods* **2025**, *14*, 2300. https://doi.org/10.3390/foods14132300

[65] Chen, Z.; Li, Q.; Yang, T.; Zhang, Y.; He, M.; Zeng, H.; Fan, H. Sequential extraction and enrichment of pesticide residues in Longan fruit by ultrasonic-assisted aqueous two-phase extraction linked to vortex-assisted dispersive liquid-liquid microextraction prior to high performance liquid chromatography analysis. *Journal of Chromatography A* **2020**, *1619*, 460929. https://doi.org/10.1016/j.chroma.2020.460929

[66] Nemati, M.; Mogaddam, M.R.A.; Farazajdeh, M.A.; Tuzen, M.; Khandaghi, J. In-situ formation/decomposition of deep eutectic solvent during solidification of

floating organic droplet-liquid-liquid microextraction method for the extraction of some antibiotics from honey prior to high performance liquid chromatography-tandem mass spectrometry. *Journal of Chromatography A* **2021**, *1660*, 462653. https://doi.org/10.1016/j.chroma.2021.462653

[67] Mandelli, A.; Guiñez, M.; Cerutti, S. Evaluation of Environmentally Relevant Nitrated and Oxygenated Polycyclic Aromatic Hydrocarbons in Honey. *Foods* **2023**, *12*(11), 2205. https://doi.org/10.3390/foods12112205

[68] Lahkak, F.E.; Taha, E.K.; Nassik, S. Detection of mycotoxins in raw milk, traditional yogurt and buttermilk in Morocco by ultra performance liquid chromatography coupled to high resolution mass spectrometry (UPLC/HRMS). *Assiut Veterinary Medical Journal* **2025**, *71*(184), 635–642. https://doi.org/10.21608/avmj.2025.312211.1344

[69] Mookantsa, S.O.; Dube, S.; Nindi, M.M. Multiclass Determination of 87 Mixed Veterinary Drugs, Pesticides and Mycotoxin Residues in Beef Muscle Samples by Ionic Liquid-Based Dispersive Liquid–Liquid Microextraction and Liquid Chromatography Tandem Mass Spectrometry. *Foods* **2025**, *14*(5), 720. https://doi.org/10.3390/foods14050720

[70] Gallocchio, F.; Moressa, A.; Zonta, G.; Angeletti, R.; Lega, F. Fast and sensitive analysis of short-and long-chain perfluoroalkyl substances in foods of animal origin. *Molecules* **2022**, *27*(22), 7899. https://doi.org/10.3390/molecules27227899
